# Linkage QTL Mapping and Genome-Wide Association Study on Resistance in Chickpea to *Pythium ultimum*

**DOI:** 10.3389/fgene.2022.945787

**Published:** 2022-08-15

**Authors:** Chiti Agarwal, Weidong Chen, Rajeev Kumar Varshney, George Vandemark

**Affiliations:** ^1^ Department of Plant Pathology, Washington State University, Pullman, WA, United States; ^2^ USDA-ARS, Grain Legume Genetics and Physiology Research Unit, Pullman, WA, United States; ^3^ Centre for Crop and Food Innovation, State Agricultural Biotechnology Centre, Murdoch University, Murdoch, WA, Australia

**Keywords:** chickpea, disease, pulses, Pythium, resistance

## Abstract

The soilborne oomycete plant pathogen *Pythium ultimum* causes seed rot and pre-emergence damping-off of chickpea (*Cicer arietinum* L*.*). The pathogen has been controlled for several decades using the fungicide metalaxyl as seed treatment but has re-emerged as a severe problem with the detection of metalaxyl-resistant isolates of the pathogen from infested fields in the United States Pacific Northwest. The objective of this study was to identify genetic markers and candidate genes associated with resistance to *P. ultimum* in an interspecific recombinant inbred line population (CRIL-7) derived from a cross between *C. reticulatum* (PI 599072) x *C. arietinum* (FLIP 84-92C) and conduct genome-wide association studies (GWAS) for disease resistance using a chickpea diversity panel consisting of 184 accessions. CRIL-7 was examined using 1029 SNP markers spanning eight linkage groups. A major QTL, “qpsd4-1,” was detected on LG 4 that explained 41.8% of phenotypic variance, and a minor QTL, “qpsd8-1,” was detected on LG8 that explained 4.5% of phenotypic variance. Seven candidate genes were also detected using composite interval mapping including several genes previously associated with disease resistance in other crop species. A total of 302,902 single nucleotide polymorphic (SNP) markers were used to determine population structure and kinship of the diversity panel. Marker–trait associations were established by employing different combinations of principal components (PC) and kinships (K) in the FarmCPU model. Genome-wide association studies detected 11 significant SNPs and seven candidate genes associated with disease resistance. SNP Ca4_1765418, detected by GWAS on chromosome 4, was located within QTL qpsd4-1 that was revealed in the interspecific CRIL-7 population. The present study provides tools to enable MAS for resistance to *P. ultimum* and identified genomic domains and candidate genes involved in the resistance of chickpea to soilborne diseases.

## Introduction

Chickpea (*Cicer arietinum* L) is one of the most historically significant field crops, being among the eight “founder crops” domesticated by Neolithic societies 8,000–12,000 years ago in the “Fertile Crescent” of present-day Iraq, Syria, and Turkey (Zohary et al., 2012). Its importance continues to this day, with more than 14.2 million tonnes of chickpea produced globally in 2019, making it the third most important pulse crop in terms of global production, after dry beans (*Phaseolus vulgaris* L.) and peas (*Pisum sativum* L.) (FAOSTAT, 2022). Chickpea is produced in more than 50 nations, with India being the largest producer, accounting for approximately 68% of global production (FAOSTAT, 2022). There are two major market classes of chickpea based on seed traits; “*Desi*” chickpea, which have a “teardrop”-shaped seed and a pigmented seed coat, and “*Kabuli*” chickpea, which has an “owl head” shape, a light beige or cream-colored seed coat, and is typically larger than the *Desi* chickpea (Toker, 2009).

In the United States, chickpea is primarily grown in dryland production systems in rotation with wheat or barley in the Pacific Northwest (Idaho and Washington) and Northern Plains (Montana and North Dakota). In 2019, more than 194,000 tonnes of chickpea were produced in the United States, with an estimated value greater than $116 million (NASS, 2022). However, several diseases challenge farmers in the United States, including Ascochyta blight caused by *Ascochyta rabiei* (Pass.) Lab. (Bayaa et al., 2011). Recently, seed rot and pre-emergent damping-off of chickpea caused by metalaxyl-resistant *P. ultimum* Trow has re-emerged as a significant disease in the Pacific Northwest.

The genus *Pythium* includes several soilborne species that cause seed and seedling diseases across a wide range of crops (Martin and Loper, 1999). *P. ultimum* causes seed rot, damping-off, and root rot in several legumes, including soybean (*Glycine* max L.), common bean (*Phaseolus vulgaris* L.), and pea (*Pisum sativum* L.) (Plaats-Niterink, 1981). Seed rot and pre-emergence damping-off of chickpea caused by *P. ultimum* were first detected in the United States in 1979 in Washington State (Kaiser and Hannan, 1983). Historically, these diseases have been controlled using pre-plant seed treatments containing metalaxyl or its stereoisomer mefenoxam (Casas et al., 1990). However, in 2014 isolates of *P. ultimum* var. *ultimum* with metalaxyl resistance were collected from unsprouted and rotten chickpea seeds obtained from a field in Washington exhibiting poor sprouting (Chen and Van Vleet, 2016). Subsequently, metalaxyl-resistant isolates of *P. ultimum* have been collected from several chickpea production fields in Idaho and Washington, where poor seedling sprouting was observed (Wang et al., 2020). Greenhouse and field tests showed that ethaboxam effectively manages metalaxyl-resistant *P. ultimum*, and commercial chickpea farmers now commonly apply a seed treatment containing both metalaxyl and ethaboxam for disease control (Wang et al., 2020). However, this increases production costs for farmers, and the effective use of ethaboxam depends on pathogen populations not developing resistance to the fungicide. Although resistance to ethaboxam in *P. ultimum* has not been detected, resistance has been detected in *P. aphanidermatum* (Edson) Fitzp. and *P. deliense* Meurs isolated from soybean (*Glycine* max L.) and dry bean (*Phaseolus vulgaris* L.), respectively. Increased production costs and concerns about the development of metalaxyl-resistant *P. ultimum* suggest that effective disease control in the future may require other approaches, including the use of disease-resistant chickpea cultivars.

Initial studies indicated that only small, dark-seeded *Desi* chickpeas were resistant to *P. ultimum* and all *Kabuli* chickpeas tested were susceptible (Kaiser and Hannan, 1983). We recently evaluated a collection of commercial chickpea cultivars and accessions from the United States National Plant Germplasm System (NPGS) and the International Crops Research Institute for the Semi-Arid Tropics (ICRISAT) for resistance to metalaxyl-resistant *P. ultimum* (Agarwal et al., 2020). The great majority of resistant accessions were *Desi* types and accessions with pigmented seed coats. Although the popular *Kabuli* cultivars ‘Sierra’ (Muehlbauer et al., 2004) and ‘Nash’ (Vandemark et al., 2015) were susceptible, three *Kabuli* accessions W625864, W625882, and W625884 were identified that were significantly more resistant than Sierra to two different isolates of metalaxyl-resistant *P. ultimum* (Agarwal et al., 2020). These results were promising because chickpea production in the United States is almost entirely composed of *Kabuli* types (Vandemark et al., 2014). Although these partially resistant accessions may be useful as parents for developing *Kabuli* cultivars with improved resistance to metalaxyl-resistant *P. ultimum*, it may also be possible to accelerate the development of resistant cultivars through the use of marker-assisted breeding approaches.

A range of genomic resources is available for chickpeas, including a draft sequence of the cultivated chickpea genome, which has an estimated size of approximately 738 Mb and contains 28,269 genes (Varshney et al., 2013). By using different marker genotyping platforms and molecular mapping approaches, significant associations have been identified between molecular markers and several diseases of chickpea, including Ascochyta blight (Tekeoglu et al., 2004; Sabbavarapu et al., 2013; Jendoubi et al., 2016; Garg et al., 2018; Mannur et al., 2019), *Fusarium* wilt (Winter et al., 2000; Sharma and Muehlbauer, 2007; Jingade and Ravikumar, 2015; Li et al., 2015; Meng et al., 2015; Mannur et al., 2019; Karadi et al., 2021), and dry root rot (Karadi et al., 2021). The objective of this study was to detect significant marker–trait associations and identify candidate genes for resistance in chickpea to metalaxyl-resistant *P. ultimum*.

## Materials and Methods

### Plant Material

A chickpea mapping population (CRIL-7) that included 177 chickpea recombinant inbred lines (RIL) derived from an interspecific cross *C. reticulatum* (PI 599072) x *C. arietinum* (FLIP 84–92C) was used to conduct QTL analysis. PI 599072 is a *Desi* type and is resistant to *P. ultimum*, whereas FLIP 84-92C is a disease-susceptible *Kabuli* type. The RILs were increased under greenhouse conditions by single seed descent to F_7_ (Tekeoglu et al., 2000). Genome-wide association study was carried out on 184 taxonomically, morphologically, and geographically diverse accessions obtained from ICRISAT, Patancheru, India. This collection included 34 *Kabuli*, 144 *Desi*, and 6 pea-shaped accessions (Upadhyaya et al., 2001).

### Disease Screening Assay and Resistance Scoring

*P. ultimum* strain PT410 (Agarwal et al., 2020) was used for all disease screening. The isolate was originally obtained from decaying chickpea seeds collected from a field in Patterson, WA, and its resistance to metalaxyl was confirmed through serial subculturing on media containing 50 ppm metalaxyl (Wang et al., 2020). The isolate was cultured and maintained on sucrose yeast extract agar in Petri plates at room temperature. CRIL-7 recombinant inbred lines along with the parental genotypes were screened for disease reaction to *P. ultimum* isolate (PT410) under controlled growth chamber conditions (12°C night–14°C days, 12 h day length) at Washington State University, Pullman, United States. Five seeds were planted in 10-cm pots containing 70 g of soil mix infested with 25,000 CFU of *P. ultimum* oospores for each entry. Myles and Sierra chickpea cultivars were used as resistant and susceptible checks, respectively (Agarwal et al., 2020). Pots were arranged in a completely randomized design. The number of seedlings that emerged from each pot was counted 14 days after planting. The experiment was repeated once.

Similarly, 184 accessions of mini-core collection from ICRISAT were evaluated for resistance to *P. ultimum* PT410 using the aforementioned methods. Again, the cultivars Sierra and Myles were included as susceptible and resistant controls. The experiment was repeated once. Results of these evaluations were previously reported (Agarwal et al., 2020).

### Statistical Analysis

Statistical analysis was conducted using JMP 14 software (MP^®^, Version <14>. SAS Institute Inc. Cary, NC, 1989–2019). Broad-sense heritability (H^2^) was calculated based on the average mean seedling emergence values from repeated experiments with R software (http://www.R-project.org/) using MME-based algorithms (Alexander and Lange, 2011).

### Linkage Group Construction and Linkage QTL Mapping

Genomic DNA was extracted from young leaves of both parents and 177 RILs. Single nucleotide polymorphic (SNP) markers were detected using the Genotyping by Sequencing (GBS) approach. The libraries from the parental lines and RILs were prepared using *Msl*I restriction enzymes and sequenced using the Illumina NextSeq 500 V^2^ to generate 150 bp paired-end reads by LGC Company (https://www.lgcgroup.com/). For processing reads, demultiplexing of all library groups was done using Illumina bcl2fastq 2.17.1.14 software, followed by demultiplexing of library groups into samples according to their barcodes. Quality trimming was done by discarding low-quality reads with a final length <20 bases, and filtered data were used to call SNPs. The filtered, high-quality reads from each sample were aligned to the chickpea reference genome (*Cicer arietinum* CDC Frontier whole-genome assembly v1.0) (Varshney et al., 2013). The variant discovery was made using Freebayes v1.0.2-16 with the parameters min-base-quality: 10; min-supportingallele-qsum: 10; read-mismatch-limit: 3; min-coverage: 5; min-alternate count: 4, excluding unobserved genotypes; and mismatch-base-quality-threshold: 10. Variant filtering was done by removing markers with missing allele calls and minor allele frequency (MAF) < 0.05. Redundant markers were excluded from the analysis by implementing the BIN function in QTL IciMaping 4.1 (Meng et al., 2015). SNP markers with highly distorted segregation ratios at probability level (*p* ≤ 0.0001) were excluded. However, SNP that were slightly distorted (0.0001 ≤ *p* ≤ 0.05) from the Mendelian ratio were included in the linkage map.

These filtered markers were used to construct linkage groups (LGs) using the “Map” function in QTL IciMaping 4.1 and were assigned numbers (LG1–LG8) based on the genomic position of SNP markers. LGs with unlinked markers were removed from further construction. The remaining SNP were grouped with a logarithm-of-odds (LOD) threshold of 9.0. Recombination counting and ordering (RECORD) and “COUNT” (number of recombination events) algorithms were used in ordering and rippling. The linkage map and the best linear unbiased predictions (BLUP) value of phenotypic data of the CRIL-7 population from repeated experiments were used for QTL analysis. QTL was detected with composite inclusive composite interval mapping of additive (ICIM-ADD) function in QTL IciMaping 4.1 (Li et al., 2007). The threshold used to declare significant QTL was the permutation test with 1,000 permutations at the 0.05 significance level. Mapping parameters to detect additive QTL were set as step = 1.0 cM and PIN = 0.001 (PIN: the largest value for entering variables in stepwise regression of residual phenotype on marker variables). Parents for trait enhancing alleles were detected using the sign of the additive effects; the positive sign denotes that trait enhancing allele is from parent PI599072, whereas the negative sign indicates that the trait-enhancing allele is from FLIP 84-92C.

### SNP Panel for GWAS

An SNP dataset from the Center of Excellence in Genomics & Systems Biology, ICRISAT that contained approximately 900,000 SNPs across 184 chickpea accessions was used for GWAS. First, SNP data were filtered by removing markers with more than 80% missing data and minor allele frequency smaller than 0.05. Duplicate markers and duplicate genotype samples in the dataset were then removed along with contigs and scaffolds using VCFtools (Hyun et al., 2008). Next, pairwise r^2^ was calculated for all SNPs across each chromosome of the chickpea genome. SNPs with significant r^2^ values (*p* < 0.001) were considered informative and were pruned using a linkage disequilibrium (LD) pruning method implemented in PLINK software v1.09 using “—indep-pairwise 50 5 0.5” command line in Linux (Purcell et al., 2007). The pruned set of SNP markers was used for association analysis.

### Population Structure and Relatedness

Population structure and kinship were estimated using ADMIXTURE (v1.23) software. The ADMIXTURE tool uses a model-based algorithm to estimate the ancestry of unrelated individuals (Alexander and Lange, 2011). The number of underlying population groups (k) was estimated from 1 to 10 using the maximum likelihood estimation approach with a fast numerical optimization algorithm. A Q-matrix file representing the least number of population groups (k) was used for GWAS. Population structure was further estimated by principal component analysis (PCA) using the PLINK function. The EMMA algorithm embedded in the GAPIT package of R software was used to account for kinship (Hyun et al., 2008; Lipka et al., 2012). Finally, a dendrogram was generated using a neighbor-joining (NJ) algorithm to assess the relationship between mini-core accessions.

### Marker–Trait Association Analysis

Association analysis was done using disease scores of the 184 chickpea accessions and 302,902 filtered SNPs. Kinship relatedness (K) was considered a random effect, and population structure based on the number of principal components (PC) that explained 25%–50% of the total phenotypic variance was considered a fixed effect. For marker–trait associations, models with different combinations of the population (PC)/admixture(Q) and family (K) structures were applied using the GAPIT package of R software: FarmCPU with Kinship (K), FarmCPU with Kinship (K) + PC(2), FarmCPU with Kinship (K) + PC(3), FarmCPU with Kinship (K) + PC(4), FarmCPU with Kinship (K) + PC(5), and FarmCPU with Kinship (K) + Admixture (Q) (Lipka et al., 2012). The best model was selected based on the mean squared difference (MSD) value between observed and expected *p*-values of all SNPs (Mamidi et al., 2011). The final Q–Q plots and Manhattan plots were created, and significant SNPs were calculated based on a *p*-value < 10^–5^ and Bonferroni cut-off (*p*-value of 0.05/(the total number of SNP markers) (i.e., 0.05/302902 = 1.65 × 10^–7^). Genes located within a 100-Kb region centered on a significant SNP were selected as candidate genes. The SNPeff tool (http://snpeff.sourceforge.net/) was used to detect genome coordinates of candidate genes and amino acid changes due to the SNPs (Cingolani et al., 2012).

## Results

### Phenotypic Evaluation for Resistance to *Pythium ultimum*.

A total of 177 CRIL-7 lines and both parents were screened for resistance to *P. ultimum* in a repeated experiment. A summary of descriptive statistics of disease reactions is presented in Supplementary Table S1. The mean of the susceptible check Sierra was 0.2 and the mean of the resistant check Myles was 4.1 in both experiments. The mean of PI 599072 was >2.5 in both experiments, indicating partial resistance to *P. ultimum*. The mean of FLIP 84-92C was ≤0.3 in both experiments, indicating susceptibility to *P. ultimum*. The means of all RILs in experiments 1 and 2 were 1.7 and 1.8, respectively. Transgressive segregants were observed among RILs in both experiments. RIL effects, experiment effects, and their interaction effects were all significant (Supplementary Table S1), with the greatest magnitude for the RIL effect and the least for the interaction effect. A broad-sense heritability estimate of 0.78 suggests that disease resistance is highly heritable (Supplementary Table S2).

### SNP-Based Interspecific Genetic Map

A total of 65,112 SNP markers were obtained by GBS on CRIL-7 population [*C. reticulatum* (PI 599072) x *C. arietinum* (FLIP 84–92C)]. After filtering, 1,029 SNP markers were used to construct a linkage map and were assigned to the eight chickpea linkage groups (LG1–LG8) (Supplementary Figure S3). The eight linkage groups covered 1,186.30 cM (Supplementary Table S4). LG 8 was the smallest linkage group with 65 markers and a length of 50.59 cM, while the largest was LG 5 with 112 markers and a total length of 281.55 cM (Supplementary Table S3). Gaps in marker coverage of LGs are due to a high proportion of distorted segregation markers among the interspecific mapping population.

### QTLs and Candidate Genes Detected by Linkage Mapping

Two QTLs were detected that were significantly associated with resistance to *P. ultimum*, QTL qpsd4-1 on LG 4, and QTL qpsd8-1 on LG 8. qpsd4-1 explained 46.5% of total variance with LOD = 25.2, whereas qpsd8-1 explained 4.5% of the total phenotypic variance with LOD = 3.3 (Table 1). qpsd4-1 had a positive additive effect value, indicating that positive alleles came from PI 599072, while the additive effect value was negative for qpsd8-1, indicating that negative alleles came from FLIP 84-92C.

**TABLE 1:** Statistical summary of QTLs for disease resistance in CRIL-7 [*C. reticulatum* (PI 599072) x *C. arietinum* (FLIP 84-92C)].

QTL	Linkage group	Position	Left marker	Right marker	LOD	Pve%	Additive effect
qpsd4-1	4	98	1793365SNPCa4	1674052SNPCa4	25.24	46.75	0.8783
qpsd8-1	8	36	2171187SNPCa8	2088222SNPCa8	3.31	4.53	–0.3109

^a^QTL, names represent the traits, the linkage group number; ^b^PVE, the percentage of phenotypic variance explained by the QTL; ^c^Additive effect with positive values shows contribution toward greater resistance, while negative values show contribution toward greater susceptibility.

The physical positions of flanking markers were used to identify putative genes associated with resistance to *P. ultimum* located within two QTLs, qpsd4-1 and qpsd8-1. *Cicer arietinum* cv. CDC Frontier (*Kabuli*) reference genome on the Pulse Crop database was used for identifying candidate genes (Varshney et al., 2013). Three candidate genes related to disease resistance were identified within the region flanking qpsd4-1 on LG 4, and four additional candidate genes were detected within the flanking qpsd8-1 region of LG 8 (Table 2).

**TABLE 2:** Candidate genes with positions and annotations from QTL analysis.

Candidate gene	^a^Ch	Physical position	Functional annotation	References
Ca_07798	4	Ca4:1741231..1747336	JmjC domain-containing protein D	Hou et al. (2015)
Ca_07797	4	Ca4:1703853..1721273	WD-repeat family	Wang et al. (2009)
Ca_07799	4	Ca4:1759881..1762270	Zinc finger protein family	Gupta et al. (2012)
Ca_02390	8	Ca8:2091020..2093474	1-aminocyclopropane-1-carboxylate synthase	(Tekeoglu et al. (2000); Tekeoglu et al. (2002))
Ca_02384	8	Ca8:2135402..2136340	AT-hook DNA-binding protein	Yadeta et al. (2011)
Ca_02383	8	Ca8:2152196..2153734	Multidrug and toxic compound extrusion (MATE) transporters	Sun et al. (2011)
Ca_02389	8	Ca8:2094005..2096765	Protein kinase family	(Martin et al., 1993; Xu et al., 2013)

a Ch. is the chromosome number of the significant SNP marker.

### Marker–Trait Association and Candidate Genes Through GWAS Analysis

For GWAS analysis, the SNP dataset on the ICRISAT accessions was filtered based on the minor allele frequencies (MAF <5%), missing data, and duplicate markers, and 229,965 SNPs were removed. Further linkage disequilibrium (LD) pruning removed 367,133 SNPs. After filtering and pruning, a total of 302,902 polymorphic SNPs remained for GWAS (Supplementary Figure S4).

**FIGURE 1:**
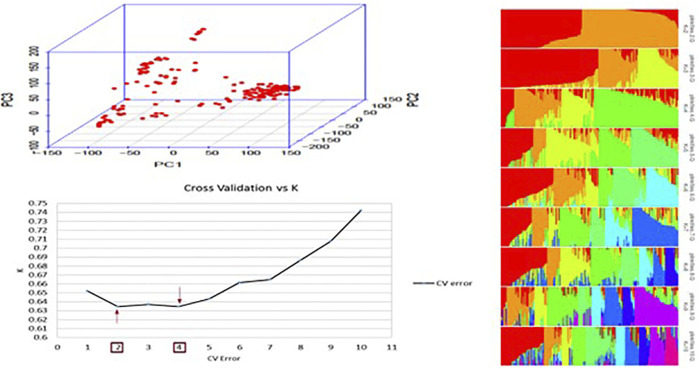
Population structure of 184 chickpea accessions. **(A)** Principal component analysis (PCA) of all accessions based on 302,902 genome-wide SNPs. PCA divided the population into two subgroups shown in the circles. **(B)** Cross-validation plot for the SNP dataset plotted using the ADMIXTURE tool. K represents the number of subpopulations, and CV is the cross-validation error. The red arrows highlight the K value with the lowest CV errors. **(C)** Bar plots for K = 2–10. Each plot was created from 184 genotypes; each single vertical line represents each genotype, and each color represents one cluster.

PCA was performed to estimate population structure. The first two principal components (PC) explained 25% of the total variance, and the first five principal components (PC) explained 50% of the total variance. Graphs plotted using the first two PC explained the distribution of genotypes within different subpopulations (Figure 1A). In ADMIXTURE analysis with a total of 10 numbers of ancestral populations, the lowest cross-validation error was observed at K = 4, followed by k = 2 (with a minimum difference) were used as cofactors for GWAS (Figure 1B). Using K = 2, the panel was split into two subpopulations corresponding to *Desi* and *Kabuli* classes (Figure 1C), while with k = 4, genotypes were grouped according to seed shapes; angular, owl, and pea-shaped.

**FIGURE 2:**
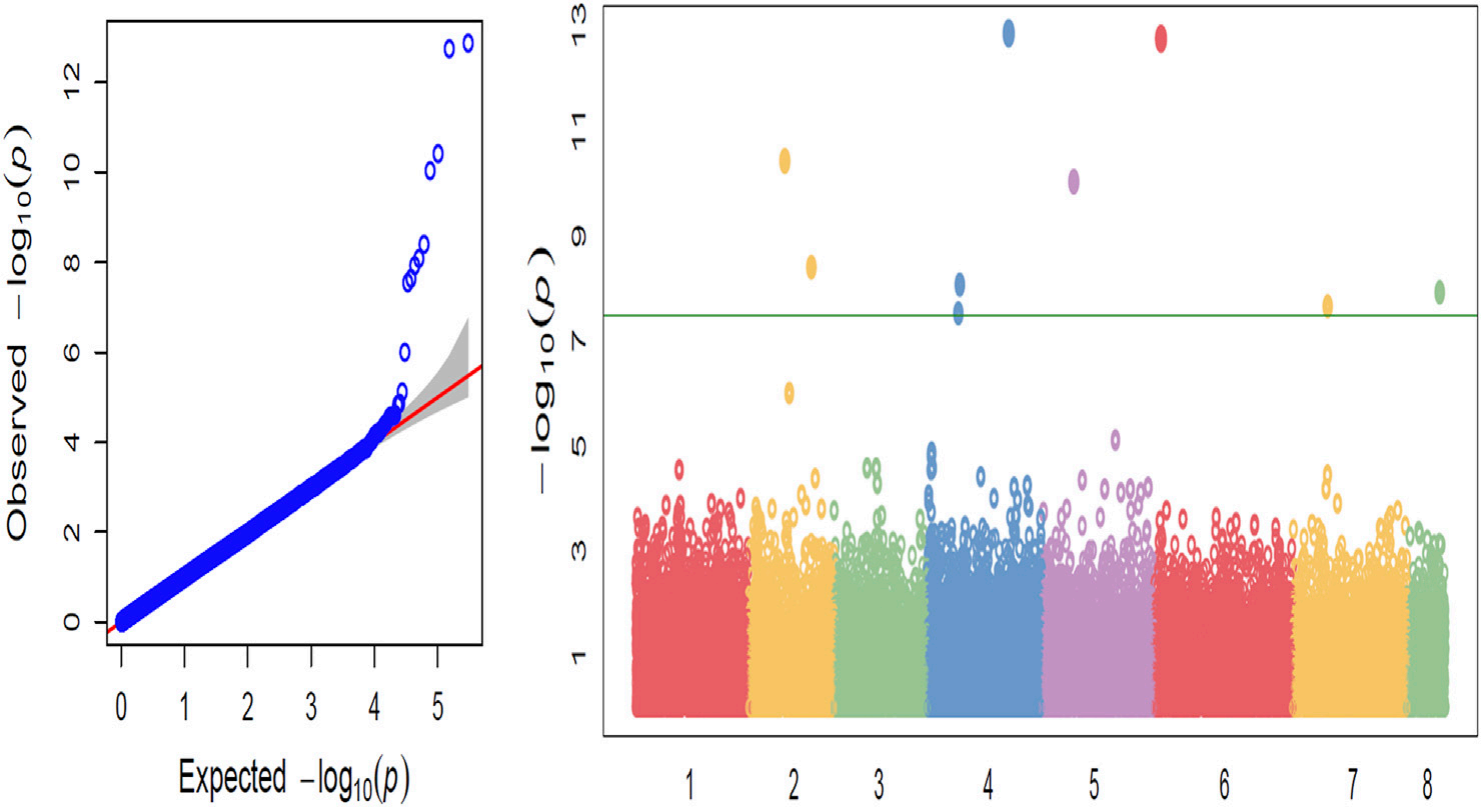
**(A)** Quantile–quantile plots illustrating the comparison between expected and observed −log10(*p*)-values. **(B)** GWAS-derived Manhattan plot showing significant *p*-values associated with disease resistance using SNPs. The *x*-axis represents the relative density of reference genome-based SNPs physically mapped on 8 chickpea chromosomes, and *y*-axis indicates the −log10(*p*)-value. Colored dots represent individual SNPs, and markers significantly associated with disease resistance are above the Bonferroni cut-off (horizontal line).

The greatest number of significant SNPs was observed on chromosome 4 (Ca4) (Figure 2). Marker Ca4_1765418 on chromosome 4 with a *p*-value of 1.65 × 10^−7^ was also detected within QTL qpsd4-1 that was revealed in the interspecific CRIL-7 population. Among the 35 significant SNPs, 29 were found in intergenic regions and 6 within genic regions (Table 3). Seven candidate genes that could be related to disease resistance were found on chromosomes 2, 4, 6, 7, and 8 within 100 kb flanking regions of significant SNPs (Table 4).

**TABLE 3:** Statistical summary of single nucleotide polymorphisms (SNPs) significantly associated with disease resistance trait.

SNP	^a^Ch	Position (bp)	Intergenic/Genic region	*p*.value	^b^MAF	FDR_Adjusted_*p*-values	Effect
Ca1_18665430	1	18665430	Intergenic	2.71E^−05^	0.315217	4.69E^−01^	0.145585
Ca1_44936836	1	44936836	Intergenic	9.94E^−05^	0.304348	8.63E^−01^	–0.20701
Ca2_15514982	2	15514982	Intergenic	3.88E^−11^	0.11413	3.92E^−06^	0.353739
Ca2_17444963	2	17444963	Genic	1.00E^−06^	0.076087	3.04E^−02^	0.221825
Ca2_22761060	2	22761060	Intergenic	8.85E^−05^	0.168478	8.19E^−01^	0.181644
Ca2_26901711	2	26901711	Intergenic	4.01E^−09^	0.057065	2.43E^−04^	0.413354
Ca2_28579479	2	28579479	Intergenic	3.99E^−05^	0.372283	5.76E^−01^	0.242365
Ca3_14221600	3	14221600	Intergenic	2.58E^−05^	0.201087	4.69E^−01^	0.197585
Ca3_18156610	3	18156610	Intergenic	2.59E^−05^	0.05163	4.69E^−01^	–0.38554
Ca3_18585784	3	18585784	Intergenic	5.18E^−05^	0.076087	6.75E^−01^	0.227405
Ca4_666303	4	666303	Intergenic	8.92E^−05^	0.30163	8.19E^−01^	0.172426
Ca4_1646485	4	1646485	Genic	2.79E^−05^	0.078804	4.69E^−01^	0.25133
Ca4_1765418	4	1765418	Intergenic	1.49E^−05^	0.350543	3.48E^−01^	0.15354
Ca4_1840434	4	1840434	Intergenic	1.35E^−05^	0.222826	3.40E^−01^	0.178898
Ca4_2249905	4	2249905	Intergenic	2.52E^−05^	0.195652	4.69E^−01^	0.197475
Ca4_13331455	4	13331455	Genic	2.93E^−08^	0.133152	9.88E^−04^	–0.33236
Ca4_14007934	4	14007934	Intergenic	8.06E^−09^	0.201087	4.07E^−04^	–0.18258
Ca4_22925858	4	22925858	Intergenic	3.88E^−05^	0.269022	5.76E^−01^	0.151793
Ca4_28639214	4	28639214	Intergenic	9.97E^−05^	0.288043	8.63E^−01^	0.150288
Ca4_34906194	4	34906194	Intergenic	1.37E^−13^	0.076087	2.67E^−08^	–0.50906
Ca4_37220588	4	37220588	Intergenic	6.10E^−05^	0.388587	6.86E^−01^	0.154591
Ca4_42835144	4	42835144	Intergenic	5.34E^−05^	0.07337	6.75E^−01^	0.229015
Ca5_13648457	5	13648457	Intergenic	9.30E^−11^	0.0625	7.04E^−06^	–0.51466
Ca5_17315201	5	17315201	Intergenic	4.23E^−05^	0.108696	5.82E^−01^	0.276777
Ca5_26889766	5	26889766	Intergenic	6.57E^−05^	0.23913	6.86E^−01^	0.182016
Ca5_31521962	5	31521962	Genic	7.48E^−06^	0.201087	2.06E^−01^	0.185133
Ca5_33795751	5	33795751	Genic	7.28E^−05^	0.067935	7.11E^−01^	0.233878
Ca5_37840504	5	37840504	Intergenic	6.28E^−05^	0.146739	6.86E^−01^	0.190138
Ca5_42306937	5	42306937	Genic	7.26E^−05^	0.092391	7.11E^−01^	0.22412
Ca5_45529647	5	45529647	Intergenic	5.82E^−05^	0.26087	6.86E^−01^	0.155,523
Ca6_2943215	6	2943215	Intergenic	1.76E^−13^	0.146739	2.67E^−08^	–0.40534
Ca7_14199535	7	14199535	Intergenic	6.34E^−05^	0.084239	6.86E-^01^	0.22165
Ca7_14818403	7	14818403	Intergenic	3.57E^−05^	0.05163	5.69E^−01^	–0.345
Ca7_14972314	7	14972314	Intergenic	2.25E^−08^	0.070652	8.51E^−04^	–0.2963
Ca8_14057710	8	14057710	Intergenic	1.19E^−08^	0.125	5.15E^−04^	–0.26813

a Ch. is the chromosome number of the significant SNP marker.

b MAF is minor allele frequency.

**TABLE 4:** Significant SNP with candidate genes and annotations from GWAS.

SNP	^a^Ch	SNP reference/alternate allele	Intergenic/Genic region	Closest candidate genes	Gene position (bp)	Functional annotation	References
Ca4_34906194	4	G/A	Intergenic	Ca_19996	34854043–34857882	Cellulose synthase–like protein	Douchkov et al. (2016)
Ca6_2943215	6	A/G	Intergenic	Ca_10436	2942150–2942952	Calmodulin-binding protein	Upadhyaya et al. (2001)
Ca2_26901711	2	C/T	Intergenic	Ca_17276	26889005–26890367	LUPR1 protein	Dong et al. (2015)
Ca2_26901711	2	C/T	Intergenic	Ca_17277	26904743–26905771	O-methyltransferase family	Yang et al. (2017)
Ca4_14007934	4	T/C	Intergenic	Ca_04625	14016692–14018353	Thiamine thiazole synthase family	(72)
Ca8_14057710	8	G/A	Intergenic	Ca_22742	14006548–14013672	Ethylene-responsive transcription factor 1-like protein	Dong et al. (2015)
Ca7_14972314	7	T/C	Intergenic	Ca_09957	14960121–14966841	Histidine kinase protein	Heo et al. (1999)

a Ch. is the chromosome number of the significant SNP marker.

## Discussion

GBS can be used to generate large-scale SNPs that are abundant in the genome allowing for the construction of high-density genetic maps and higher statistical power in association studies (Spencer et al., 2009; Yang et al., 2017). The CRIL-7 population has been used previously to develop linkage maps. These maps covered 981.6 cM and 1,174.4 cM across 9 linkage groups based on isozyme, inter simple sequence repeat (ISSR) loci, and RAPD markers (Santra et al., 2000b; Tekeoglu et al., 2002). In the present study, SNPs were used to resolve eight linkage groups spanning 1,186.30 cM.

### QTLs and Candidate Genes Detected by Linkage Mapping

A major QTL for resistance to metalaxyl-resistant *P. ultimum*, qpsd4-1, was detected on LG 4 (Table 1). Previously, CRIL-7 has been evaluated for Ascochyta blight resistance, and QTLs associated with disease resistance were detected on LG 1, LG 4, and LG 8 (Santra et al., 2000a; Tekeoglu et al., 2004; Kumar et al., 2018). Two significant clusters of QTLs (QTLAR1 and QTLAR2) associated with resistance to Ascochyta blight have also been detected on LG 4 based on an analysis of RILs from a cross of cultivars Amit and ICCV 96029 (Deokar et al., 2019). Additionally, two QTLs (AB-Q-SR-4-1 and AB-Q-SR-4-2) on LG 4 associated with Ascochyta blight disease resistance were detected using mapping population (C 214’ × ‘ILC 3279) (Gisi and Sierotzki, 2008). These results and our detection of a major QTL for disease resistance on LG 4 suggest that genes for resistance to diverse chickpea pathogens are located on LG 4.

A total of seven genes associated with disease resistance were found in the QTL region on LG 4 and LG 8. Three candidate genes, Ca_07798, Ca_07797, and Ca_07799, were identified in QTL qpsd4-1. Ca_07798 is a JmjC domain-containing protein D, which is a group of histone lysine demethylases. This protein positively regulates rice defense against bacterial blight pathogen *Xanthomonas oryzae p*v. oryzae by epigenetically suppressing negative defense regulator H3K4me2/3 (Hou et al., 2015). The Ca_07797 gene belongs to a WD-repeat family that is involved in plant innate immune signaling pathway. Studies on maize showed that WDR-containing TTG1 protein–induced resistance against leaf blights (Ibraheem et al., 2010; Ibraheem et al., 2015). In tobacco, the interaction of TTG1-WDR with an elicitin protein (ParA1) from a pathogenic oomycete *Phytophthora parasitica* var. *nicotianae*-activated plant immune responses, including the generation of reactive oxygen species and programmed cell death (Wang et al., 2009). The Ca_07799 gene belongs to a zinc finger protein family that has been shown to play a critical role in disease resistance across many plant species (Gupta et al., 2012).

The Ca_02390 gene found on LG 8 encodes for 1-aminocyclopropane-1-carboxylate synthase. This enzyme initiates the conversion of S-adenosyl-l-methionine (SAM) into 1-aminocyclopropane-1-carboxylate (ACC), which is the precursor of ethylene and acts as a signaling molecule to regulate plant growth and reduce stress response (Polko and Kieber, 2019). The role of ACC and ethylene biosynthesis in plant defense against bacterial pathogens, including *Erwinia carotovora subsp.* carotovora and *Pseudomonas syringae* have been studied in *Arabidopsis thaliana* (L.) Heynh mutants where plants with reduced ACC production showed greater disease susceptibility (Norman-Setterblad et al., 2000; Guan et al., 2015). Recently, upregulation of genes involved in ethylene biosynthesis was detected in resistant apple seedling reactions to infection by *P. ultimum* (Zhu and Saltzgiver, 2020). Ca_02384 on LG 8 encodes an AT-hook DNA-binding protein that binds to minor groove DNA and alters gene expression. Genes such as AHL19 encoding an AT-hook DNA-binding protein are associated with enhanced disease resistance in *A. thaliana* to Verticillium wilt caused by *V. dahliae*, *V. albo-atrum*, *and V. longisporum* (Yadeta et al., 2011). The Ca_02383 gene on LG 8 belongs to the family of multidrug and toxic compound extrusion (MATE) transporters associated with plant disease resistance during pathogen interaction. The expression of MATE genes in plants is induced by pathogen attack (Sun et al., 2011). Members of the MATE family such as enhanced disease susceptibility 5 (EDS5) and activated disease susceptibility 1 (ADS1) function as negative regulators of plant immune systems by reducing basal resistance during pathogen interaction or by negatively regulating the accumulation of salicylic acid and pathogenesis-related 1 (PR1) gene expression (Nawrath et al., 2002; Sun et al., 2011). Another candidate gene on LG 8, Ca_02389, belongs to the protein kinase family. Members of this family have also been shown to be upregulated in response to pathogens, for example, *Xanthomonas oryzae pv. oryzicola* (*Xoc*) in *Oryza sativa* and *Pseudomonas syringae* pv tomato (*pto*) in *Solanum lycopersicum* (Martin et al., 1993; Xu et al., 2013). Recently, GWAS of common bean identified several candidate genes associated with resistance to *P. ultimum*, including genes for protein kinase superfamily proteins and MAPK/ERK kinase 1 (Dramadri et al., 2020).

### GWAS Analysis of Resistance in Chickpea to *P. ultimum*

Different statistical models were deployed using FarmCPU to assess population structure and kinship in the chickpea diversity panel. A combination of these models using FarmCPU separates a mixed linear model (MLM) into a random effect and a fixed-effect model, which reduces false positives and false negatives caused by kinship and population structure and gives highly significant SNP markers (Lipka et al., 2012). The best model was selected based on MSD value (Supplementary Table S5), with a low MSD value indicating less deviation from the expected distribution of *p*-values, signifying lower type I error of the selected model. This study identified a total of seven candidate gene mapping to 11 loci associated with resistance in chickpea to *P. ultimum*. PCA and ADMIXTURE analyses revealed two major groups within the core collections (Figure 1) corresponding to *Desi* and *Kabuli* market classes.

GWAS identified many SNPs associated with disease-resistance–related traits. Gene Ca_19996 encodes cellulose synthase–like protein, which inhibits the progress of the fungal penetration peg during powdery mildew infection caused by *Blumeria graminis f. sp. hordei (Bgh)* in barley (*Hordeum vulgare* L.) (Douchkov et al., 2016). Gene Ca_09957 encodes a histidine kinase protein involved in seed maturation and disease resistance against fungal and bacterial pathogens (Pham et al., 2012). Ca_17277 belongs to the O-methyltransferase family of enzymes that play a significant role in plant stress and disease resistance. Studies on corn (*Zea mays* L.) and wheat (*Triticum aestivum* L.) demonstrated that caffeoyl-CoA O-methyltransferase conferred resistance against southern leaf blight, gray leaf spot, and sharp eyespot disease, respectively (Yang et al., 2017; Wang et al., 2018). Ca_04625 encodes a thiamine thiazole synthase that increases resistance to fungal pathogens by enhancing anti-oxidative capacity and inducing systemic acquired resistance (SAR) in diverse plant species, including *Oryza sativa* L. *A. thaliana* (L.) Heynh*.*, *Nicotiana sp*., and *Cucumis sativus* L. (Goyer, 2010). Ca_22742 encodes an ethylene-responsive transcription factor 1–like protein. Ethylene-responsive transcription factors play a critical role in the plant defense system by regulating pathogenesis-related (PR) gene expression, including effectors GmERF5 and GmERF113, and contribute to resistance against root and seed rot caused by Oomycete pathogens, including *Phytopthora nicotianae* and *Py. sojae* (Goyer, 2010; Yang et al., 2017). The Ca_17276 gene encodes LUPR1 protein that is upregulated in response to the *Hyaloperonospora parasitica* (*LURP*) cluster in part of the *A. thaliana*. The LUPR1 gene has been associated with resistance to oomycetes *Hyaloperonospora parasitica* and *Py. infestans* (Dong et al., 2015). The Ca_10436 gene encodes a calmodulin-binding protein, which has activated and enhanced resistance to a broad spectrum of pathogens in *Nicotiana tabacum* and *A. thaliana* (Zhao et al., 2017)*.* Additionally, calmodulin-binding proteins in *A. thaliana* and *Hordeum vulgare* have been shown to confer resistance to powdery mildew by interacting with MLO (powdery mildew-resistance gene o) protein (Heo et al., 1999).

## Conclusion

In this study, we used an interspecific chickpea population to identify one major and one minor QTL associated with resistance to *P. ultimum*. We also identified 35 SNPs and 14 candidate genes associated with disease resistance based on the GWAS of a chickpea diversity panel. SNP Ca4_1765418, detected by GWAS on chromosome 4, was located within QTL qpsd4-1 that was revealed in the interspecific CRIL-7 population. These findings suggest this region of the genome should be examined more closely to identify genes conditioning disease resistance. Significant QTLs must be validated in different chickpea populations before the markers can be widely used for breeding. The present study provides tools to enable MAS for resistance to *P. ultimum* and identified genomic domains and candidate genes involved in the resistance of chickpea to soilborne diseases.

## Data Availability

The set of 184 genotypes used for GWAS analysis in the present study is part of 300 genotypes that were sequenced in an earlier study (Varshney et al., 2013). Sequencing data for 184 genotypes as part of 300 genotypes have been reported in the earlier study. These data are available in the NCBI under accession code SRA: SRP096939; BioProject: PRJNA362278, and in the CNSA (https://db.cngb.org/cnsa/) of CNGBdb with accession code CNP0000370.
