# Supplementary material for: Linkage QTL Mapping and Genome-Wide Association Study on Resistance in Chickpea to Pythium ultimum
Source: Front Genet. 2022 Aug 15;13:945787. doi: 10.3389/fgene.2022.945787 (PMC9420999; doi:10.3389/fgene.2022.945787)
Supplement: Supplementary file 1 [file DataSheet1.docx]

# Supplementary Table 1. Phenotypic variation of CRIL-7 in parents and RIL population across both experiments for the number of seedlings that emerge 14 days after planting in soil infested with *Pythium ultimum*^1^.

|  | Parents | | | CRIL-7 population | | | | | | | |
| --- | --- | --- | --- | --- | --- | --- | --- | --- | --- | --- | --- |
| Environment | PI 599072 | FLIP 84-92C | | Range | | | Mean± SD | Variance | Skewness | | Shapiro Wilk-test |
| Experiment 1 | 3 | | 0.3 | | 0-5 | 1.8±1.5 | | 2.3431 | | 0.48 | 0.8896 |
| Experiment 2 | 2.6 | | 0.3 | | 0-5 | 1.7±1.2 | | 1.685 | | 0.42 | 0.9188 |

^1^Five seeds were planted in each pot. Four replicate pots were tested for each entry in each experiment.

# Supplementary Table 2. Analysis of variance (ANOVA) and broad-sense heritability for number of seedlings^a^ that emerge 14 days after planting in soil infested with *P. ultimum* PT410 for 177 Chickpea RILs [*C. reticulatum* (PI 599072) x *C. arietinum* (FLIP 84-92C)].

| Source | DF | Sum of square | Mean square | F-value | Pr>F | H^2^ (%) |
| --- | --- | --- | --- | --- | --- | --- |
| RIL | 176 | 1,794.24 | 10.1945 | 12.1894 | 0.0000 | 78.2 |
| Experiment | 1 | 5.88 | 5.8767 | 7.0266 | 0.0082 |  |
| RIL x Experiment | 176 | 332.62 | 1.8899 | 2.2597 | 0.0000 |  |

^a^Five seeds were planted in each pot infested with 25,000 oospores of *P. ultimum* PT410. Four replicate pots were tested for each RIL.

# Supplementary Table 3. Summary of 8 linkage groups (LG) of a genetic map for CRIL-7 [*C. reticulatum* (PI 599072) x *C. arietinum* (FLIP 84-92C)].

| Linkage group ID | Total number of SNP markers | Length (cM) |
| --- | --- | --- |
| LG 1 | 126 | 151.35 |
| LG 2 | 129 | 177.89 |
| LG 3 | 114 | 67.02 |
| LG 4 | 153 | 103.76 |
| LG 5 | 114 | 281.55 |
| LG 6 | 158 | 190.17 |
| LG 7 | 171 | 164.62 |
| LG 8 | 66 | 50.6 |
| Whole Genome | 1029 | 1186.96 |

# Supplementary Table 4. Descriptive statistics for the genetic linkage map of the CRIL7 [*C. reticulatum* (PI 599072) x *C. arietinum* (FLIP 84-92C)] population.

| SNP Marker | Linkage Group | Position (cM) | ^a^Markers with Segregation Distortion |
| --- | --- | --- | --- |
| 46835069SNPCa1 | 1 | 0 | * |
| 8821388SNPCa1 | 1 | 0.28 | * |
| 8821312SNPCa1 | 1 | 0.57 | * |
| 8977141SNPCa1 | 1 | 1.14 | * |
| 9017940SNPCa1 | 1 | 1.42 | * |
| 9074622SNPCa1 | 1 | 1.71 | * |
| 9094667SNPCa1 | 1 | 1.99 | * |
| 9216171SNPCa1 | 1 | 3.83 | * |
| 9246538SNPCa1 | 1 | 4.13 | * |
| 9546761SNPCa1 | 1 | 5.35 | * |
| 11130184SNPCa1 | 1 | 18.9 | * |
| 11290981SNPCa1 | 1 | 19.48 | * |
| 11291723SNPCa1 | 1 | 19.77 | * |
| 11435198SNPCa1 | 1 | 20.37 |  |
| 11674475SNPCa1 | 1 | 20.95 |  |
| 11816561SNPCa1 | 1 | 21.32 |  |
| 11710811SNPCa1 | 1 | 22.08 | * |
| 11853089SNPCa1 | 1 | 22.69 | * |
| 11969952SNPCa1 | 1 | 23.3 | * |
| 12250628SNPCa1 | 1 | 23.88 |  |
| 12316897SNPCa1 | 1 | 24.63 |  |
| 12611695SNPCa1 | 1 | 25.77 | * |
| 12411345SNPCa1 | 1 | 26.34 | * |
| 12562060SNPCa1 | 1 | 26.62 | * |
| 12685854SNPCa1 | 1 | 27.8 |  |
| 13072801SNPCa1 | 1 | 29.28 |  |
| 13072868SNPCa1 | 1 | 29.56 |  |
| 13228816SNPCa1 | 1 | 29.85 |  |
| 13360113SNPCa1 | 1 | 30.13 |  |
| 13288460SNPCa1 | 1 | 30.42 |  |
| 13409344SNPCa1 | 1 | 30.7 |  |
| 13410786SNPCa1 | 1 | 30.98 |  |
| 13772321SNPCa1 | 1 | 31.56 |  |
| 13910837SNPCa1 | 1 | 32.13 |  |
| 14093105SNPCa1 | 1 | 33.28 | * |
| 14310142SNPCa1 | 1 | 33.57 | * |
| 14306460SNPCa1 | 1 | 33.86 | * |
| 14373874SNPCa1 | 1 | 34.72 |  |
| 14419356SNPCa1 | 1 | 35.01 |  |
| 14490144SNPCa1 | 1 | 35.31 |  |
| 14670240SNPCa1 | 1 | 35.62 |  |
| 15270936SNPCa1 | 1 | 37.64 |  |
| 15151893SNPCa1 | 1 | 38.02 |  |
| 15229719SNPCa1 | 1 | 38.6 |  |
| 15529246SNPCa1 | 1 | 39.76 | * |
| 15687629SNPCa1 | 1 | 40.04 | * |
| 15690449SNPCa1 | 1 | 40.33 | * |
| 15925559SNPCa1 | 1 | 40.92 | * |
| 16006473SNPCa1 | 1 | 41.52 | * |
| 16035882SNPCa1 | 1 | 41.81 | * |
| 16228257SNPCa1 | 1 | 42.39 | * |
| 16310103SNPCa1 | 1 | 42.67 | * |
| 16371243SNPCa1 | 1 | 43.83 | * |
| 16448769SNPCa1 | 1 | 44.12 | * |
| 16448813SNPCa1 | 1 | 44.4 | * |
| 16600663SNPCa1 | 1 | 44.69 | * |
| 16569564SNPCa1 | 1 | 44.97 | * |
| 16789905SNPCa1 | 1 | 45.26 | * |
| 16877855SNPCa1 | 1 | 45.54 | * |
| 41813581SNPCa1 | 1 | 45.83 | * |
| 41918523SNPCa1 | 1 | 46.11 | * |
| 41813574SNPCa1 | 1 | 46.68 | * |
| 41972744SNPCa1 | 1 | 46.98 | * |
| 31486SNP | 1 | 47.88 |  |
| 47989160SNPCa1 | 1 | 48.45 | * |
| 47938498SNPCa1 | 1 | 48.74 | * |
| 47057521SNPCa1 | 1 | 49.02 |  |
| 41055710SNPCa1 | 1 | 49.59 |  |
| 22370860SNPCa1 | 1 | 50.46 |  |
| 21615504SNPCa1 | 1 | 50.74 |  |
| 21591086SNPCa1 | 1 | 51.02 |  |
| 19672609SNPCa1 | 1 | 51.6 |  |
| 18816605SNPCa1 | 1 | 51.88 |  |
| 18042120SNPCa1 | 1 | 52.74 |  |
| 18254018SNPCa1 | 1 | 53.03 |  |
| 17552751SNPCa1 | 1 | 53.6 |  |
| 17359686SNPCa1 | 1 | 54.46 |  |
| 8506097SNPCa1 | 1 | 55.04 |  |
| 8384600SNPCa1 | 1 | 55.91 |  |
| 8266287SNPCa1 | 1 | 56.49 |  |
| 7978723SNPCa1 | 1 | 57.09 |  |
| 7631100SNPCa1 | 1 | 57.69 |  |
| 7489078SNPCa1 | 1 | 58.26 |  |
| 7326115SNPCa1 | 1 | 59.13 |  |
| 7265726SNPCa1 | 1 | 59.7 |  |
| 6935925SNPCa1 | 1 | 60.56 |  |
| 6559343SNPCa1 | 1 | 61.8 |  |
| 6328468SNPCa1 | 1 | 63.05 |  |
| 5978521SNPCa1 | 1 | 63.33 |  |
| 5753268SNPCa1 | 1 | 64.5 |  |
| 5630589SNPCa1 | 1 | 65.07 |  |
| 5411550SNPCa1 | 1 | 65.36 |  |
| 5242536SNPCa1 | 1 | 65.97 |  |
| 4889456SNPCa1 | 1 | 66.26 |  |
| 4423068SNPCa1 | 1 | 67.72 |  |
| 4232608SNPCa1 | 1 | 68.58 |  |
| 4283909SNPCa1 | 1 | 69.16 |  |
| 4105315SNPCa1 | 1 | 69.73 |  |
| 3686448SNPCa1 | 1 | 70.89 |  |
| 3446007SNPCa1 | 1 | 72.04 |  |
| 3359148SNPCa1 | 1 | 72.33 |  |
| 3255353SNPCa1 | 1 | 72.92 |  |
| 3186894SNPCa1 | 1 | 73.21 |  |
| 2939598SNPCa1 | 1 | 73.79 |  |
| 2718359SNPCa1 | 1 | 74.07 |  |
| 2890522SNPCa1 | 1 | 74.36 |  |
| 2608340SNPCa1 | 1 | 74.94 |  |
| 2523882SNPCa1 | 1 | 75.81 |  |
| 2295317SNPCa1 | 1 | 76.38 |  |
| 2158927SNPCa1 | 1 | 77.67 |  |
| 2060711SNPCa1 | 1 | 78.67 |  |
| 1974714SNPCa1 | 1 | 79.29 |  |
| 1824051SNPCa1 | 1 | 82.38 |  |
| 1782788SNPCa1 | 1 | 82.7 |  |
| 1796785SNPCa1 | 1 | 83.35 | * |
| 1738267SNPCa1 | 1 | 83.66 | * |
| 1703262SNPCa1 | 1 | 84.29 |  |
| 1681511SNPCa1 | 1 | 84.59 |  |
| 1528011SNPCa1 | 1 | 84.89 |  |
| 1338345SNPCa1 | 1 | 86.06 |  |
| 1032591SNPCa1 | 1 | 86.34 |  |
| 1011981SNPCa1 | 1 | 86.63 |  |
| 910019SNPCa1 | 1 | 87.21 |  |
| 558802SNPCa1 | 1 | 88.07 |  |
| 103483SNPCa1 | 1 | 89.23 |  |
| 204199SNPCa1 | 1 | 89.85 |  |
| 45033417SNPCa1 | 1 | 151.07 |  |
| 44986637SNPCa1 | 1 | 151.35 |  |
| 1630619SNPCa2 | 2 | 0 |  |
| 1820936SNPCa2 | 2 | 1.16 |  |
| 1845570SNPCa2 | 2 | 1.77 |  |
| 1906118SNPCa2 | 2 | 2.37 |  |
| 2041175SNPCa2 | 2 | 3.83 |  |
| 2126842SNPCa2 | 2 | 4.41 |  |
| 2117225SNPCa2 | 2 | 4.69 |  |
| 2225778SNPCa2 | 2 | 6.14 |  |
| 2358436SNPCa2 | 2 | 6.44 |  |
| 2470115SNPCa2 | 2 | 6.74 |  |
| 2470047SNPCa2 | 2 | 7.03 |  |
| 2514149SNPCa2 | 2 | 8.21 | * |
| 2557859SNPCa2 | 2 | 9.09 | * |
| 2632216SNPCa2 | 2 | 9.66 |  |
| 2632234SNPCa2 | 2 | 9.94 | * |
| 2748973SNPCa2 | 2 | 12.38 |  |
| 2892961SNPCa2 | 2 | 13.27 |  |
| 2902404SNPCa2 | 2 | 13.84 | * |
| 3013638SNPCa2 | 2 | 14.42 | * |
| 3013584SNPCa2 | 2 | 14.71 | * |
| 3082156SNPCa2 | 2 | 15.29 | * |
| 3155720SNPCa2 | 2 | 15.57 | * |
| 3225491SNPCa2 | 2 | 15.9 |  |
| 3521864SNPCa2 | 2 | 17.28 | * |
| 3481582SNPCa2 | 2 | 17.57 | * |
| 3673307SNPCa2 | 2 | 17.86 | * |
| 3736925SNPCa2 | 2 | 19.2 |  |
| 3793095SNPCa2 | 2 | 19.65 | * |
| 3838067SNPCa2 | 2 | 19.94 | * |
| 3838139SNPCa2 | 2 | 20.22 | * |
| 3923444SNPCa2 | 2 | 20.51 | * |
| 3923441SNPCa2 | 2 | 20.79 | * |
| 3988455SNPCa2 | 2 | 21.08 | * |
| 4128016SNPCa2 | 2 | 21.36 | * |
| 74092SNP | 2 | 25.36 | * |
| 171352SNP | 2 | 25.64 | * |
| 5610755SNPCa2 | 2 | 27.4 | * |
| 5875933SNPCa2 | 2 | 27.97 | * |
| 6159327SNPCa2 | 2 | 28.26 | * |
| 6405211SNPCa2 | 2 | 28.54 |  |
| 6541098SNPCa2 | 2 | 28.83 |  |
| 6868989SNPCa2 | 2 | 29.4 |  |
| 6888481SNPCa2 | 2 | 29.69 |  |
| 6965114SNPCa2 | 2 | 29.97 |  |
| 9943404SNPCa2 | 2 | 30.84 |  |
| 8618837SNPCa2 | 2 | 31.7 |  |
| 8715332SNPCa2 | 2 | 31.98 |  |
| 9266095SNPCa2 | 2 | 32.56 |  |
| 10264986SNPCa2 | 2 | 34.02 |  |
| 10358987SNPCa2 | 2 | 34.89 |  |
| 11480834SNPCa2 | 2 | 35.17 |  |
| 10358906SNPCa2 | 2 | 35.74 |  |
| 11737876SNPCa2 | 2 | 36.33 |  |
| 601730SNP | 2 | 38.45 |  |
| 105913SNP | 2 | 39.09 |  |
| 64749SNP | 2 | 46.45 |  |
| 24831706SNPCa2 | 2 | 52.19 |  |
| 25213714SNPCa2 | 2 | 52.47 |  |
| 25540875SNPCa2 | 2 | 52.76 |  |
| 26559252SNPCa2 | 2 | 53.91 |  |
| 15460SNP | 2 | 54.78 |  |
| 28224044SNPCa2 | 2 | 56.87 |  |
| 28337390SNPCa2 | 2 | 57.45 |  |
| 28372662SNPCa2 | 2 | 58.03 |  |
| 29539558SNPCa2 | 2 | 59.22 |  |
| 29759720SNPCa2 | 2 | 59.8 |  |
| 29411195SNPCa2 | 2 | 60.38 |  |
| 30353716SNPCa2 | 2 | 60.67 |  |
| 30353599SNPCa2 | 2 | 60.95 |  |
| 29925930SNPCa2 | 2 | 61.24 |  |
| 63130SNP | 2 | 62.12 |  |
| 30374892SNPCa2 | 2 | 63.33 |  |
| 30377798SNPCa2 | 2 | 63.63 |  |
| 30546442SNPCa2 | 2 | 64.51 |  |
| 30631543SNPCa2 | 2 | 64.81 |  |
| 30736226SNPCa2 | 2 | 65.1 |  |
| 30684829SNPCa2 | 2 | 65.39 |  |
| 30696612SNPCa2 | 2 | 65.68 |  |
| 30910784SNPCa2 | 2 | 66.86 |  |
| 30910681SNPCa2 | 2 | 67.44 |  |
| 31819639SNPCa2 | 2 | 68.02 |  |
| 31912270SNPCa2 | 2 | 68.89 |  |
| 31974840SNPCa2 | 2 | 69.5 |  |
| 32098689SNPCa2 | 2 | 72.37 |  |
| 32270933SNPCa2 | 2 | 73.54 |  |
| 32260639SNPCa2 | 2 | 74.71 |  |
| 33003361SNPCa2 | 2 | 75 |  |
| 32838741SNPCa2 | 2 | 75.29 |  |
| 33140495SNPCa2 | 2 | 75.88 |  |
| 33193337SNPCa2 | 2 | 76.19 |  |
| 33329454SNPCa2 | 2 | 76.51 |  |
| 33445603SNPCa2 | 2 | 77.09 |  |
| 33674161SNPCa2 | 2 | 78.26 |  |
| 33786880SNPCa2 | 2 | 78.83 |  |
| 33764565SNPCa2 | 2 | 79.12 |  |
| 34307631SNPCa2 | 2 | 79.7 |  |
| 5461SNP | 2 | 79.99 |  |
| 34354584SNPCa2 | 2 | 80.27 |  |
| 34607816SNPCa2 | 2 | 81.14 |  |
| 34432630SNPCa2 | 2 | 81.43 |  |
| 13843SNP | 2 | 82.01 |  |
| 34747085SNPCa2 | 2 | 82.58 |  |
| 35105961SNPCa2 | 2 | 84.96 |  |
| 35172354SNPCa2 | 2 | 85.25 |  |
| 35347613SNPCa2 | 2 | 85.54 |  |
| 35450796SNPCa2 | 2 | 85.82 |  |
| 35442357SNPCa2 | 2 | 86.4 |  |
| 35473093SNPCa2 | 2 | 87.89 |  |
| 35538116SNPCa2 | 2 | 88.19 |  |
| 35563859SNPCa2 | 2 | 88.47 |  |
| 35563944SNPCa2 | 2 | 89.35 |  |
| 35642656SNPCa2 | 2 | 89.93 |  |
| 35611284SNPCa2 | 2 | 90.22 |  |
| 35756729SNPCa2 | 2 | 91.39 |  |
| 35849561SNPCa2 | 2 | 93.47 |  |
| 35890728SNPCa2 | 2 | 94.04 |  |
| 36025716SNPCa2 | 2 | 94.34 |  |
| 36555409SNPCa2 | 2 | 96.85 |  |
| 36261003SNPCa2 | 2 | 97.13 |  |
| 1255190SNPCa2 | 2 | 177.61 |  |
| 1258289SNPCa2 | 2 | 177.89 |  |
| 39678426SNPCa3 | 3 | 0 |  |
| 39849483SNPCa3 | 3 | 1.79 |  |
| 39678336SNPCa3 | 3 | 2.08 |  |
| 39583648SNPCa3 | 3 | 2.67 |  |
| 39467438SNPCa3 | 3 | 2.96 |  |
| 39383840SNPCa3 | 3 | 3.24 |  |
| 39349993SNPCa3 | 3 | 3.53 |  |
| 39120858SNPCa3 | 3 | 4.69 |  |
| 39084609SNPCa3 | 3 | 4.98 |  |
| 39016269SNPCa3 | 3 | 5.55 |  |
| 38887369SNPCa3 | 3 | 6.42 |  |
| 38829721SNPCa3 | 3 | 6.7 |  |
| 38505251SNPCa3 | 3 | 6.99 |  |
| 38386005SNPCa3 | 3 | 7.86 |  |
| 38238577SNPCa3 | 3 | 9.34 |  |
| 38334427SNPCa3 | 3 | 10.21 |  |
| 38108302SNPCa3 | 3 | 10.79 |  |
| 37892943SNPCa3 | 3 | 11.96 |  |
| 37843506SNPCa3 | 3 | 12.25 |  |
| 37527248SNPCa3 | 3 | 12.82 |  |
| 37266903SNPCa3 | 3 | 13.11 |  |
| 37143873SNPCa3 | 3 | 13.4 |  |
| 37072041SNPCa3 | 3 | 13.68 |  |
| 36885191SNPCa3 | 3 | 14.26 |  |
| 36793314SNPCa3 | 3 | 14.85 |  |
| 36711012SNPCa3 | 3 | 15.14 |  |
| 36676946SNPCa3 | 3 | 15.72 | * |
| 36640653SNPCa3 | 3 | 16.04 | * |
| 36194086SNPCa3 | 3 | 17.39 | * |
| 36122934SNPCa3 | 3 | 17.68 | * |
| 36122902SNPCa3 | 3 | 17.97 | * |
| 35931116SNPCa3 | 3 | 19.14 | * |
| 35722794SNPCa3 | 3 | 20.01 |  |
| 35617004SNPCa3 | 3 | 20.65 |  |
| 35709471SNPCa3 | 3 | 20.97 |  |
| 35605195SNPCa3 | 3 | 21.55 | * |
| 35542312SNPCa3 | 3 | 22.12 | * |
| 35395420SNPCa3 | 3 | 22.41 | * |
| 35218370SNPCa3 | 3 | 22.99 | * |
| 34804419SNPCa3 | 3 | 23.57 | * |
| 34498138SNPCa3 | 3 | 25.08 | * |
| 34399493SNPCa3 | 3 | 25.38 | * |
| 34260810SNPCa3 | 3 | 25.97 | * |
| 33184206SNPCa3 | 3 | 26.27 | * |
| 33125315SNPCa3 | 3 | 26.58 | * |
| 32938407SNPCa3 | 3 | 26.87 | * |
| 32909841SNPCa3 | 3 | 27.16 | * |
| 32704586SNPCa3 | 3 | 27.44 | * |
| 31692638SNPCa3 | 3 | 28.02 | * |
| 31572256SNPCa3 | 3 | 28.88 | * |
| 31442828SNPCa3 | 3 | 29.45 | * |
| 30957791SNPCa3 | 3 | 30.31 |  |
| 30752417SNPCa3 | 3 | 30.6 |  |
| 29998368SNPCa3 | 3 | 31.77 |  |
| 29888676SNPCa3 | 3 | 32.64 |  |
| 29575825SNPCa3 | 3 | 34.73 |  |
| 29497129SNPCa3 | 3 | 35.69 |  |
| 29456338SNPCa3 | 3 | 36 |  |
| 29401508SNPCa3 | 3 | 36.29 |  |
| 29231646SNPCa3 | 3 | 36.57 |  |
| 29191998SNPCa3 | 3 | 37.15 |  |
| 28809642SNPCa3 | 3 | 37.73 |  |
| 28662829SNPCa3 | 3 | 38.31 |  |
| 28471862SNPCa3 | 3 | 39.17 |  |
| 28295659SNPCa3 | 3 | 39.46 |  |
| 27767294SNPCa3 | 3 | 40.04 |  |
| 27682541SNPCa3 | 3 | 40.34 |  |
| 27538010SNPCa3 | 3 | 40.62 |  |
| 27348518SNPCa3 | 3 | 41.5 |  |
| 27193828SNPCa3 | 3 | 42.37 |  |
| 27193736SNPCa3 | 3 | 42.95 |  |
| 26789025SNPCa3 | 3 | 43.81 |  |
| 26678488SNPCa3 | 3 | 44.38 |  |
| 26276766SNPCa3 | 3 | 46.14 |  |
| 26270311SNPCa3 | 3 | 46.42 |  |
| 26178726SNPCa3 | 3 | 46.7 |  |
| 25494149SNPCa3 | 3 | 46.99 |  |
| 25391086SNPCa3 | 3 | 47.56 |  |
| 25221977SNPCa3 | 3 | 48.42 |  |
| 24935201SNPCa3 | 3 | 48.71 |  |
| 24485630SNPCa3 | 3 | 49.28 |  |
| 24119279SNPCa3 | 3 | 49.56 | * |
| 24095537SNPCa3 | 3 | 49.85 |  |
| 23977705SNPCa3 | 3 | 50.13 |  |
| 23885370SNPCa3 | 3 | 50.42 |  |
| 23854742SNPCa3 | 3 | 51.29 | * |
| 23455889SNPCa3 | 3 | 51.87 |  |
| 22848405SNPCa3 | 3 | 53.32 |  |
| 22602750SNPCa3 | 3 | 53.61 |  |
| 22200981SNPCa3 | 3 | 54.47 |  |
| 22139830SNPCa3 | 3 | 54.75 |  |
| 22096635SNPCa3 | 3 | 55.08 |  |
| 22023707SNPCa3 | 3 | 55.42 |  |
| 21809789SNPCa3 | 3 | 56.31 |  |
| 21777125SNPCa3 | 3 | 56.6 |  |
| 21542160SNPCa3 | 3 | 56.88 |  |
| 21352272SNPCa3 | 3 | 57.45 |  |
| 21032611SNPCa3 | 3 | 58.02 |  |
| 20948866SNPCa3 | 3 | 58.9 |  |
| 20813823SNPCa3 | 3 | 59.19 |  |
| 20738661SNPCa3 | 3 | 59.48 |  |
| 20311457SNPCa3 | 3 | 59.77 |  |
| 20244725SNPCa3 | 3 | 60.06 |  |
| 19722658SNPCa3 | 3 | 60.34 |  |
| 197934SNP | 3 | 61.26 |  |
| 17970894SNPCa3 | 3 | 61.86 |  |
| 17568857SNPCa3 | 3 | 62.15 |  |
| 54188SNP | 3 | 62.55 | * |
| 4542295SNPCa3 | 3 | 64.6 |  |
| 352045SNPCa3 | 3 | 66.13 |  |
| 228321SNPCa3 | 3 | 66.73 |  |
| 135225SNP | 3 | 67.02 |  |
| 49139711SNPCa4 | 4 | 0 |  |
| 48996450SNPCa4 | 4 | 0.3 |  |
| 48814549SNPCa4 | 4 | 0.61 |  |
| 48535430SNPCa4 | 4 | 1.19 |  |
| 48370525SNPCa4 | 4 | 2.07 |  |
| 48268253SNPCa4 | 4 | 2.36 |  |
| 48157759SNPCa4 | 4 | 5.06 |  |
| 48144539SNPCa4 | 4 | 5.34 |  |
| 48079571SNPCa4 | 4 | 6.19 | * |
| 47635991SNPCa4 | 4 | 7.47 |  |
| 47471838SNPCa4 | 4 | 8.34 |  |
| 47446975SNPCa4 | 4 | 8.63 |  |
| 47330196SNPCa4 | 4 | 9.2 |  |
| 47236870SNPCa4 | 4 | 9.49 |  |
| 47189819SNPCa4 | 4 | 10.65 |  |
| 46745752SNPCa4 | 4 | 11.28 |  |
| 46686692SNPCa4 | 4 | 11.9 |  |
| 46636253SNPCa4 | 4 | 12.19 |  |
| 46499041SNPCa4 | 4 | 13.06 |  |
| 46433692SNPCa4 | 4 | 13.34 |  |
| 46334676SNPCa4 | 4 | 13.63 |  |
| 46127765SNPCa4 | 4 | 14.55 |  |
| 45941843SNPCa4 | 4 | 16.09 | * |
| 45832282SNPCa4 | 4 | 16.44 |  |
| 45616450SNPCa4 | 4 | 17.9 |  |
| 45582648SNPCa4 | 4 | 18.2 |  |
| 45475491SNPCa4 | 4 | 18.79 |  |
| 45365383SNPCa4 | 4 | 19.37 |  |
| 45273065SNPCa4 | 4 | 19.66 |  |
| 45151853SNPCa4 | 4 | 21.67 | * |
| 45072910SNPCa4 | 4 | 22.66 |  |
| 44986054SNPCa4 | 4 | 22.99 |  |
| 44874275SNPCa4 | 4 | 23.66 | * |
| 44849271SNPCa4 | 4 | 23.95 | * |
| 44591134SNPCa4 | 4 | 24.82 |  |
| 44418203SNPCa4 | 4 | 25.73 | * |
| 44362494SNPCa4 | 4 | 26.03 | * |
| 44184697SNPCa4 | 4 | 26.33 |  |
| 44172586SNPCa4 | 4 | 27.22 | * |
| 44069892SNPCa4 | 4 | 27.51 | * |
| 43996951SNPCa4 | 4 | 27.8 |  |
| 41998332SNPCa4 | 4 | 28.97 |  |
| 41809371SNPCa4 | 4 | 30.49 |  |
| 41673686SNPCa4 | 4 | 31.09 |  |
| 41513770SNPCa4 | 4 | 31.67 |  |
| 41332866SNPCa4 | 4 | 31.97 |  |
| 41265417SNPCa4 | 4 | 32.56 |  |
| 41188995SNPCa4 | 4 | 33.2 |  |
| 41110276SNPCa4 | 4 | 33.52 |  |
| 41003713SNPCa4 | 4 | 34.42 |  |
| 40753526SNPCa4 | 4 | 35.62 |  |
| 40753571SNPCa4 | 4 | 36.23 |  |
| 40596988SNPCa4 | 4 | 37.14 |  |
| 40278069SNPCa4 | 4 | 38.01 |  |
| 39858706SNPCa4 | 4 | 38.88 |  |
| 38835698SNPCa4 | 4 | 39.45 |  |
| 38495096SNPCa4 | 4 | 40.33 |  |
| 38461868SNPCa4 | 4 | 40.62 |  |
| 38423008SNPCa4 | 4 | 41.2 |  |
| 38288600SNPCa4 | 4 | 42.06 |  |
| 36422826SNPCa4 | 4 | 42.64 |  |
| 37095611SNPCa4 | 4 | 44.16 |  |
| 37095548SNPCa4 | 4 | 45.38 |  |
| 25126949SNPCa4 | 4 | 46.55 |  |
| 21749007SNPCa4 | 4 | 49.25 |  |
| 20481069SNPCa4 | 4 | 50.41 |  |
| 20076753SNPCa4 | 4 | 50.7 |  |
| 19595253SNPCa4 | 4 | 50.99 |  |
| 17675863SNPCa4 | 4 | 53.05 |  |
| 17603613SNPCa4 | 4 | 53.33 |  |
| 17156597SNPCa4 | 4 | 53.62 |  |
| 17003599SNPCa4 | 4 | 53.91 |  |
| 16832082SNPCa4 | 4 | 55.09 |  |
| 16759279SNPCa4 | 4 | 55.38 |  |
| 15997317SNPCa4 | 4 | 56.48 |  |
| 16336942SNPCa4 | 4 | 57.21 |  |
| 15670120SNPCa4 | 4 | 57.79 |  |
| 15476785SNPCa4 | 4 | 58.07 |  |
| 15394470SNPCa4 | 4 | 58.65 |  |
| 15144595SNPCa4 | 4 | 58.93 |  |
| 14870870SNPCa4 | 4 | 59.22 |  |
| 14840245SNPCa4 | 4 | 60.09 |  |
| 14689048SNPCa4 | 4 | 60.37 |  |
| 14520548SNPCa4 | 4 | 61.24 |  |
| 14415224SNPCa4 | 4 | 61.82 |  |
| 14184416SNPCa4 | 4 | 62.4 |  |
| 14090005SNPCa4 | 4 | 62.69 |  |
| 13407692SNPCa4 | 4 | 63.86 |  |
| 13306332SNPCa4 | 4 | 64.14 |  |
| 13062060SNPCa4 | 4 | 64.72 |  |
| 12768894SNPCa4 | 4 | 65.01 |  |
| 12563222SNPCa4 | 4 | 65.3 |  |
| 12488581SNPCa4 | 4 | 66.18 |  |
| 12359013SNPCa4 | 4 | 67.96 |  |
| 12257195SNPCa4 | 4 | 68.25 |  |
| 11989597SNPCa4 | 4 | 68.55 |  |
| 11948409SNPCa4 | 4 | 68.84 |  |
| 11746928SNPCa4 | 4 | 69.71 |  |
| 10988250SNPCa4 | 4 | 70.29 |  |
| 10848397SNPCa4 | 4 | 70.57 |  |
| 10772564SNPCa4 | 4 | 70.86 |  |
| 10538951SNPCa4 | 4 | 71.73 |  |
| 10017169SNPCa4 | 4 | 73.2 |  |
| 9876424SNPCa4 | 4 | 73.5 |  |
| 9669125SNPCa4 | 4 | 73.81 |  |
| 9406989SNPCa4 | 4 | 74.38 |  |
| 9325234SNPCa4 | 4 | 74.97 |  |
| 9241446SNPCa4 | 4 | 75.26 |  |
| 8928750SNPCa4 | 4 | 75.55 |  |
| 8737317SNPCa4 | 4 | 76.44 |  |
| 8667126SNPCa4 | 4 | 76.72 |  |
| 8430098SNPCa4 | 4 | 77.3 |  |
| 8346101SNPCa4 | 4 | 77.88 |  |
| 8226051SNPCa4 | 4 | 78.75 |  |
| 7799524SNPCa4 | 4 | 79.91 |  |
| 7607593SNPCa4 | 4 | 81.5 |  |
| 7415995SNPCa4 | 4 | 81.81 |  |
| 7066896SNPCa4 | 4 | 82.39 |  |
| 6986852SNPCa4 | 4 | 82.97 |  |
| 6955006SNPCa4 | 4 | 83.26 |  |
| 6907911SNPCa4 | 4 | 84.16 |  |
| 6826471SNPCa4 | 4 | 84.46 |  |
| 6704584SNPCa4 | 4 | 85.04 |  |
| 6506368SNPCa4 | 4 | 85.33 |  |
| 6315028SNPCa4 | 4 | 86.22 |  |
| 6193996SNPCa4 | 4 | 86.51 |  |
| 5937417SNPCa4 | 4 | 86.79 |  |
| 5576068SNPCa4 | 4 | 87.37 |  |
| 4871871SNPCa4 | 4 | 88.24 |  |
| 4505137SNPCa4 | 4 | 89.12 |  |
| 4475041SNPCa4 | 4 | 89.41 |  |
| 4310499SNPCa4 | 4 | 89.7 |  |
| 4228468SNPCa4 | 4 | 90.29 |  |
| 4124151SNPCa4 | 4 | 90.63 |  |
| 4003139SNPCa4 | 4 | 91.32 |  |
| 3941967SNPCa4 | 4 | 91.92 |  |
| 3693120SNPCa4 | 4 | 92.52 |  |
| 3729561SNPCa4 | 4 | 92.81 |  |
| 3641174SNPCa4 | 4 | 93.39 |  |
| 3441194SNPCa4 | 4 | 93.68 |  |
| 3197790SNPCa4 | 4 | 94.28 |  |
| 3139994SNPCa4 | 4 | 94.91 |  |
| 3040907SNPCa4 | 4 | 95.55 |  |
| 2815667SNPCa4 | 4 | 96.12 |  |
| 2517306SNPCa4 | 4 | 96.7 |  |
| 2248186SNPCa4 | 4 | 96.99 |  |
| 1848359SNPCa4 | 4 | 97.57 |  |
| 1793365SNPCa4 | 4 | 97.86 |  |
| 1674052SNPCa4 | 4 | 98.74 |  |
| 1254172SNPCa4 | 4 | 100.51 |  |
| 1083178SNPCa4 | 4 | 100.8 |  |
| 831459SNPCa4 | 4 | 102.25 |  |
| 485160SNPCa4 | 4 | 103.11 |  |
| 30195906SNPCa5 | 5 | 0 |  |
| 30344371SNPCa5 | 5 | 0.9 |  |
| 30380156SNPCa5 | 5 | 1.19 |  |
| 30582395SNPCa5 | 5 | 1.47 | * |
| 30692369SNPCa5 | 5 | 2.05 | * |
| 30797531SNPCa5 | 5 | 2.34 | * |
| 31158252SNPCa5 | 5 | 2.63 | * |
| 31177369SNPCa5 | 5 | 2.91 | * |
| 31266062SNPCa5 | 5 | 3.2 | * |
| 31714863SNPCa5 | 5 | 4.98 | * |
| 32098088SNPCa5 | 5 | 6.15 | * |
| 32108738SNPCa5 | 5 | 6.73 | * |
| 32459453SNPCa5 | 5 | 7.6 | * |
| 32508227SNPCa5 | 5 | 8.18 | * |
| 32673677SNPCa5 | 5 | 8.47 | * |
| 32591343SNPCa5 | 5 | 8.77 | * |
| 32867236SNPCa5 | 5 | 9.37 | * |
| 33129053SNPCa5 | 5 | 10.54 | * |
| 1930SNP | 5 | 10.83 | * |
| 33415802SNPCa5 | 5 | 11.41 | * |
| 33828832SNPCa5 | 5 | 12.29 | * |
| 33947095SNPCa5 | 5 | 12.87 | * |
| 34301815SNPCa5 | 5 | 13.16 | * |
| 34436395SNPCa5 | 5 | 13.45 | * |
| 34705681SNPCa5 | 5 | 14.03 | * |
| 34973237SNPCa5 | 5 | 14.9 | * |
| 35360747SNPCa5 | 5 | 15.77 | * |
| 35513332SNPCa5 | 5 | 16.35 | * |
| 36422909SNPCa5 | 5 | 19.05 |  |
| 36769027SNPCa5 | 5 | 19.62 |  |
| 36985024SNPCa5 | 5 | 19.91 |  |
| 37279563SNPCa5 | 5 | 20.77 | * |
| 37540562SNPCa5 | 5 | 21.06 | * |
| 37776871SNPCa5 | 5 | 22.53 |  |
| 37874782SNPCa5 | 5 | 23.11 |  |
| 37983823SNPCa5 | 5 | 23.68 | * |
| 38061045SNPCa5 | 5 | 23.97 | * |
| 48071754SNPCa5 | 5 | 24.26 | * |
| 47755373SNPCa5 | 5 | 25.42 |  |
| 47591829SNPCa5 | 5 | 25.71 |  |
| 47519879SNPCa5 | 5 | 25.99 |  |
| 47353833SNPCa5 | 5 | 26.28 |  |
| 47100320SNPCa5 | 5 | 26.56 |  |
| 47049791SNPCa5 | 5 | 27.15 |  |
| 46943243SNPCa5 | 5 | 28.63 |  |
| 46815425SNPCa5 | 5 | 28.92 |  |
| 46517350SNPCa5 | 5 | 29.84 |  |
| 46328086SNPCa5 | 5 | 30.13 |  |
| 46173074SNPCa5 | 5 | 30.71 |  |
| 45947641SNPCa5 | 5 | 31.01 |  |
| 45924047SNPCa5 | 5 | 31.32 |  |
| 45811278SNPCa5 | 5 | 31.89 |  |
| 45521296SNPCa5 | 5 | 33.06 |  |
| 45419723SNPCa5 | 5 | 33.63 |  |
| 45120254SNPCa5 | 5 | 33.92 |  |
| 45030350SNPCa5 | 5 | 34.31 |  |
| 44975166SNPCa5 | 5 | 35.1 |  |
| 44686574SNPCa5 | 5 | 36.27 |  |
| 44560128SNPCa5 | 5 | 36.56 |  |
| 44489652SNPCa5 | 5 | 36.84 |  |
| 44388416SNPCa5 | 5 | 37.14 |  |
| 44347329SNPCa5 | 5 | 37.43 |  |
| 43892698SNPCa5 | 5 | 39.83 |  |
| 43569991SNPCa5 | 5 | 40.7 |  |
| 43158151SNPCa5 | 5 | 40.98 |  |
| 42884075SNPCa5 | 5 | 42.14 |  |
| 42422319SNPCa5 | 5 | 43.01 |  |
| 42413900SNPCa5 | 5 | 43.3 |  |
| 42041788SNPCa5 | 5 | 44.47 |  |
| 41961347SNPCa5 | 5 | 44.76 |  |
| 41343191SNPCa5 | 5 | 45.63 |  |
| 40981124SNPCa5 | 5 | 46.5 |  |
| 40762282SNPCa5 | 5 | 48.56 |  |
| 40567223SNPCa5 | 5 | 48.84 |  |
| 40534454SNPCa5 | 5 | 49.77 |  |
| 40427020SNPCa5 | 5 | 50.07 |  |
| 40243369SNPCa5 | 5 | 50.64 |  |
| 40029939SNPCa5 | 5 | 50.93 |  |
| 39999735SNPCa5 | 5 | 51.21 |  |
| 38792244SNPCa5 | 5 | 52.99 |  |
| 38681617SNPCa5 | 5 | 53.56 |  |
| 29910495SNPCa5 | 5 | 53.85 |  |
| 29348330SNPCa5 | 5 | 54.5 |  |
| 29634258SNPCa5 | 5 | 54.82 |  |
| 29284195SNPCa5 | 5 | 55.39 |  |
| 183950SNP | 5 | 56.84 |  |
| 1217343SNPCa5 | 5 | 57.13 |  |
| 28847248SNPCa5 | 5 | 57.75 |  |
| 28731379SNPCa5 | 5 | 58.06 |  |
| 28686071SNPCa5 | 5 | 58.34 |  |
| 28536727SNPCa5 | 5 | 58.92 |  |
| 28318683SNPCa5 | 5 | 59.2 |  |
| 28235527SNPCa5 | 5 | 59.49 |  |
| 28032004SNPCa5 | 5 | 60.36 |  |
| 27853889SNPCa5 | 5 | 60.65 |  |
| 27631192SNPCa5 | 5 | 60.93 |  |
| 27515220SNPCa5 | 5 | 61.8 |  |
| 27480661SNPCa5 | 5 | 62.37 |  |
| 27141512SNPCa5 | 5 | 62.95 |  |
| 26199620SNPCa5 | 5 | 63.82 |  |
| 26058526SNPCa5 | 5 | 64.1 |  |
| 25927353SNPCa5 | 5 | 64.97 |  |
| 24119520SNPCa5 | 5 | 66.43 |  |
| 10379SNP | 5 | 67.3 |  |
| 132277SNP | 5 | 69.04 | * |
| 147973SNP | 5 | 72.02 |  |
| 70114SNP | 5 | 72.6 |  |
| 70012SNP | 5 | 73.18 |  |
| 9521700SNPCa5 | 5 | 75.59 |  |
| 9711170SNPCa5 | 5 | 75.88 |  |
| 10147075SNPCa5 | 5 | 76.17 |  |
| 7607006SNPCa5 | 5 | 76.75 |  |
| 7569503SNPCa5 | 5 | 77.34 |  |
| 483693SNPCa5 | 5 | 120.32 |  |
| 6730SNPCa5 | 5 | 120.6 |  |
| 30055378SNPCa5 | 5 | 201.08 |  |
| 43139617SNPCa5 | 5 | 281.55 | * |
| 203594SNPCa6 | 6 | 0 | * |
| 253385SNPCa6 | 6 | 0.87 | * |
| 821369SNPCa6 | 6 | 1.44 | * |
| 1809214SNPCa6 | 6 | 4.12 | * |
| 1780353SNPCa6 | 6 | 4.42 | * |
| 1922354SNPCa6 | 6 | 5.01 | * |
| 2074979SNPCa6 | 6 | 5.6 | * |
| 2095741SNPCa6 | 6 | 5.88 | * |
| 2257121SNPCa6 | 6 | 6.45 | * |
| 2389673SNPCa6 | 6 | 7.91 | * |
| 2408109SNPCa6 | 6 | 8.77 | * |
| 2631922SNPCa6 | 6 | 9.34 | * |
| 2719517SNPCa6 | 6 | 9.91 | * |
| 2847526SNPCa6 | 6 | 10.2 | * |
| 3294567SNPCa6 | 6 | 11.06 | * |
| 3477049SNPCa6 | 6 | 11.65 | * |
| 3851716SNPCa6 | 6 | 12.24 | * |
| 3891908SNPCa6 | 6 | 13.11 | * |
| 4048852SNPCa6 | 6 | 13.39 | * |
| 4175687SNPCa6 | 6 | 13.67 | * |
| 4528327SNPCa6 | 6 | 13.96 | * |
| 4762515SNPCa6 | 6 | 14.24 | * |
| 5200672SNPCa6 | 6 | 14.82 | * |
| 5257495SNPCa6 | 6 | 15.12 | * |
| 5368419SNPCa6 | 6 | 15.43 | * |
| 5629552SNPCa6 | 6 | 16 | * |
| 5962791SNPCa6 | 6 | 16.28 | * |
| 6145060SNPCa6 | 6 | 16.86 | * |
| 6236836SNPCa6 | 6 | 17.14 | * |
| 6250184SNPCa6 | 6 | 17.46 | * |
| 6426807SNPCa6 | 6 | 18.42 | * |
| 6954924SNPCa6 | 6 | 18.71 | * |
| 7217976SNPCa6 | 6 | 19.58 | * |
| 7284719SNPCa6 | 6 | 19.87 | * |
| 7486733SNPCa6 | 6 | 20.73 | * |
| 7520940SNPCa6 | 6 | 21.31 | * |
| 7631185SNPCa6 | 6 | 22.22 | * |
| 7663250SNPCa6 | 6 | 23.44 | * |
| 7865434SNPCa6 | 6 | 24.3 | * |
| 8134381SNPCa6 | 6 | 24.87 | * |
| 8134426SNPCa6 | 6 | 25.15 | * |
| 8688380SNPCa6 | 6 | 25.44 | * |
| 8816488SNPCa6 | 6 | 25.88 | * |
| 9111081SNPCa6 | 6 | 27.22 | * |
| 9414390SNPCa6 | 6 | 28.38 | * |
| 10263097SNPCa6 | 6 | 30.75 | * |
| 8119SNP | 6 | 31.37 | * |
| 10654443SNPCa6 | 6 | 31.68 | * |
| 10779801SNPCa6 | 6 | 32.98 | * |
| 12904976SNPCa6 | 6 | 33.62 | * |
| 12951355SNPCa6 | 6 | 34.19 | * |
| 20437SNP | 6 | 34.48 | * |
| 13188302SNPCa6 | 6 | 35.08 | * |
| 13289000SNPCa6 | 6 | 35.65 | * |
| 13601667SNPCa6 | 6 | 36.51 | * |
| 14036739SNPCa6 | 6 | 36.8 | * |
| 14478389SNPCa6 | 6 | 37.68 | * |
| 15156201SNPCa6 | 6 | 39.79 | * |
| 15315923SNPCa6 | 6 | 40.65 | * |
| 15387623SNPCa6 | 6 | 40.94 | * |
| 15534815SNPCa6 | 6 | 41.22 | * |
| 15686499SNPCa6 | 6 | 41.79 | * |
| 15659826SNPCa6 | 6 | 42.08 | * |
| 15821562SNPCa6 | 6 | 43.24 | * |
| 16390144SNPCa6 | 6 | 43.82 | * |
| 16581051SNPCa6 | 6 | 44.83 | * |
| 16765438SNPCa6 | 6 | 45.16 | * |
| 17114699SNPCa6 | 6 | 46.08 | * |
| 17824971SNPCa6 | 6 | 47 | * |
| 17509274SNPCa6 | 6 | 47.59 | * |
| 18652972SNPCa6 | 6 | 48.77 | * |
| 19259284SNPCa6 | 6 | 50.24 | * |
| 19411976SNPCa6 | 6 | 50.82 | * |
| 49973SNP | 6 | 51.99 | * |
| 78300SNP | 6 | 52.57 | * |
| 20605077SNPCa6 | 6 | 53.48 | * |
| 20649967SNPCa6 | 6 | 53.78 | * |
| 20831395SNPCa6 | 6 | 54.07 | * |
| 21045117SNPCa6 | 6 | 54.42 | * |
| 21178321SNPCa6 | 6 | 54.78 | * |
| 21351524SNPCa6 | 6 | 55.06 | * |
| 21633582SNPCa6 | 6 | 56.31 | * |
| 21689394SNPCa6 | 6 | 56.93 | * |
| 21864004SNPCa6 | 6 | 57.22 | * |
| 21964117SNPCa6 | 6 | 57.79 | * |
| 22496839SNPCa6 | 6 | 58.67 | * |
| 23258695SNPCa6 | 6 | 59.83 | * |
| 23384070SNPCa6 | 6 | 60.12 |  |
| 23436216SNPCa6 | 6 | 60.4 | * |
| 23487676SNPCa6 | 6 | 60.69 | * |
| 23535837SNPCa6 | 6 | 60.97 | * |
| 23637212SNPCa6 | 6 | 61.26 | * |
| 24254182SNPCa6 | 6 | 62.49 | * |
| 24406117SNPCa6 | 6 | 63.09 | * |
| 24571435SNPCa6 | 6 | 63.96 | * |
| 52036631SNPCa6 | 6 | 64.83 | * |
| 51531520SNPCa6 | 6 | 65.4 | * |
| 51489515SNPCa6 | 6 | 65.74 | * |
| 51200009SNPCa6 | 6 | 66.4 | * |
| 50370725SNPCa6 | 6 | 66.98 | * |
| 49784627SNPCa6 | 6 | 67.55 | * |
| 92976SNP | 6 | 67.85 | * |
| 49525569SNPCa6 | 6 | 68.78 |  |
| 47541395SNPCa6 | 6 | 69.08 | * |
| 192300SNP | 6 | 69.95 | * |
| 30917189SNPCa6 | 6 | 71.71 | * |
| 29355390SNPCa6 | 6 | 73.17 | * |
| 29245652SNPCa6 | 6 | 73.46 | * |
| 28979606SNPCa6 | 6 | 74.03 | * |
| 28913025SNPCa6 | 6 | 74.33 | * |
| 28094264SNPCa6 | 6 | 75.22 | * |
| 27892418SNPCa6 | 6 | 75.51 | * |
| 27756603SNPCa6 | 6 | 75.79 | * |
| 27588284SNPCa6 | 6 | 76.14 | * |
| 27559351SNPCa6 | 6 | 76.52 | * |
| 27541845SNPCa6 | 6 | 76.83 | * |
| 27464189SNPCa6 | 6 | 77.12 | * |
| 27427379SNPCa6 | 6 | 77.41 | * |
| 27082087SNPCa6 | 6 | 78.27 | * |
| 27023543SNPCa6 | 6 | 78.56 | * |
| 26891835SNPCa6 | 6 | 79.71 |  |
| 26811637SNPCa6 | 6 | 80 |  |
| 26681329SNPCa6 | 6 | 80.29 |  |
| 26517675SNPCa6 | 6 | 80.87 |  |
| 26470795SNPCa6 | 6 | 81.16 |  |
| 26360533SNPCa6 | 6 | 81.73 |  |
| 54349008SNPCa6 | 6 | 82.3 |  |
| 54167470SNPCa6 | 6 | 84.36 |  |
| 54154274SNPCa6 | 6 | 84.65 |  |
| 54091079SNPCa6 | 6 | 84.93 |  |
| 53482430SNPCa6 | 6 | 85.21 |  |
| 53588048SNPCa6 | 6 | 86.38 |  |
| 53619087SNPCa6 | 6 | 87.54 |  |
| 53925518SNPCa6 | 6 | 90.29 |  |
| 57345404SNPCa6 | 6 | 91.17 |  |
| 57390289SNPCa6 | 6 | 91.45 |  |
| 57472697SNPCa6 | 6 | 92.03 |  |
| 57546815SNPCa6 | 6 | 92.32 |  |
| 57597174SNPCa6 | 6 | 92.92 |  |
| 57624902SNPCa6 | 6 | 93.21 |  |
| 57673799SNPCa6 | 6 | 94.7 |  |
| 57858657SNPCa6 | 6 | 97.74 |  |
| 57898197SNPCa6 | 6 | 100.79 |  |
| 57937252SNPCa6 | 6 | 102.05 |  |
| 57937298SNPCa6 | 6 | 102.34 |  |
| 58190494SNPCa6 | 6 | 104.17 |  |
| 58176500SNPCa6 | 6 | 104.46 |  |
| 58234994SNPCa6 | 6 | 105.06 |  |
| 58375046SNPCa6 | 6 | 106.23 |  |
| 58633162SNPCa6 | 6 | 108.62 |  |
| 58864383SNPCa6 | 6 | 109.19 |  |
| 58940075SNPCa6 | 6 | 109.48 |  |
| 59095492SNPCa6 | 6 | 110.06 |  |
| 11339816SNPCa6 | 6 | 174.19 |  |
| 11348132SNPCa6 | 6 | 174.48 |  |
| 57158047SNPCa6 | 6 | 186.38 | * |
| 57186224SNPCa6 | 6 | 186.68 | * |
| 57115321SNPCa6 | 6 | 188.16 | * |
| 57108225SNPCa6 | 6 | 188.74 | * |
| 56795702SNPCa6 | 6 | 189.31 | * |
| 56619552SNPCa6 | 6 | 189.89 | * |
| 56531926SNPCa6 | 6 | 190.17 | * |
| 49713SNPCa7 | 7 | 0 |  |
| 692179SNPCa7 | 7 | 1.17 |  |
| 692221SNPCa7 | 7 | 1.46 |  |
| 803692SNPCa7 | 7 | 2.04 |  |
| 852224SNPCa7 | 7 | 2.32 |  |
| 1035700SNPCa7 | 7 | 2.9 |  |
| 992984SNPCa7 | 7 | 3.19 |  |
| 1107617SNPCa7 | 7 | 3.48 |  |
| 1315708SNPCa7 | 7 | 4.63 |  |
| 1396683SNPCa7 | 7 | 6.14 |  |
| 1389068SNPCa7 | 7 | 6.44 |  |
| 1507833SNPCa7 | 7 | 6.73 |  |
| 1681053SNPCa7 | 7 | 7.02 |  |
| 1586613SNPCa7 | 7 | 7.31 |  |
| 1499773SNPCa7 | 7 | 7.6 |  |
| 1798944SNPCa7 | 7 | 8.47 |  |
| 2161186SNPCa7 | 7 | 9.05 |  |
| 2318823SNPCa7 | 7 | 9.63 |  |
| 2489650SNPCa7 | 7 | 9.92 |  |
| 2489558SNPCa7 | 7 | 10.2 |  |
| 2694661SNPCa7 | 7 | 10.78 |  |
| 2775480SNPCa7 | 7 | 12.25 |  |
| 2730363SNPCa7 | 7 | 12.55 |  |
| 2827245SNPCa7 | 7 | 13.14 |  |
| 2827685SNPCa7 | 7 | 13.43 |  |
| 2902828SNPCa7 | 7 | 13.74 |  |
| 2948109SNPCa7 | 7 | 14.36 |  |
| 3188333SNPCa7 | 7 | 14.93 |  |
| 3188399SNPCa7 | 7 | 15.22 |  |
| 3507297SNPCa7 | 7 | 16.1 |  |
| 3657176SNPCa7 | 7 | 16.67 |  |
| 3713909SNPCa7 | 7 | 16.96 |  |
| 3862899SNPCa7 | 7 | 17.55 |  |
| 3862932SNPCa7 | 7 | 17.83 |  |
| 4283375SNPCa7 | 7 | 19.03 |  |
| 4327871SNPCa7 | 7 | 19.33 | * |
| 4405383SNPCa7 | 7 | 19.61 |  |
| 4432289SNPCa7 | 7 | 19.91 | * |
| 4577858SNPCa7 | 7 | 20.83 | * |
| 4578475SNPCa7 | 7 | 21.12 | * |
| 4651668SNPCa7 | 7 | 21.42 | * |
| 4651479SNPCa7 | 7 | 21.72 | * |
| 4893068SNPCa7 | 7 | 22.3 | * |
| 5251130SNPCa7 | 7 | 22.88 | * |
| 5278057SNPCa7 | 7 | 23.16 | * |
| 5266764SNPCa7 | 7 | 23.49 |  |
| 5356827SNPCa7 | 7 | 24.16 | * |
| 6541834SNPCa7 | 7 | 25.03 | * |
| 6228680SNPCa7 | 7 | 25.32 |  |
| 6147862SNPCa7 | 7 | 25.61 |  |
| 6530881SNPCa7 | 7 | 25.89 |  |
| 6637779SNPCa7 | 7 | 26.84 | * |
| 6793164SNPCa7 | 7 | 27.16 | * |
| 7100343SNPCa7 | 7 | 28.02 | * |
| 7183500SNPCa7 | 7 | 28.89 | * |
| 7258164SNPCa7 | 7 | 29.47 | * |
| 7258177SNPCa7 | 7 | 29.76 | * |
| 7484249SNPCa7 | 7 | 30.33 |  |
| 7385706SNPCa7 | 7 | 30.62 | * |
| 7422749SNPCa7 | 7 | 30.91 | * |
| 7763451SNPCa7 | 7 | 32.38 | * |
| 7892698SNPCa7 | 7 | 32.67 | * |
| 8088693SNPCa7 | 7 | 34.78 | * |
| 8072404SNPCa7 | 7 | 35.07 | * |
| 8135109SNPCa7 | 7 | 35.38 | * |
| 8171031SNPCa7 | 7 | 35.7 | * |
| 8340621SNPCa7 | 7 | 36.89 | * |
| 8524693SNPCa7 | 7 | 38.39 |  |
| 8511097SNPCa7 | 7 | 38.67 |  |
| 8784202SNPCa7 | 7 | 39.25 | * |
| 8682477SNPCa7 | 7 | 39.54 |  |
| 8635853SNPCa7 | 7 | 39.83 |  |
| 8915530SNPCa7 | 7 | 40.7 | * |
| 8952403SNPCa7 | 7 | 40.99 | * |
| 9151705SNPCa7 | 7 | 41.27 | * |
| 9732833SNPCa7 | 7 | 41.85 | * |
| 825329SNP | 7 | 42.42 | * |
| 708856SNP | 7 | 42.99 |  |
| 708977SNP | 7 | 43.28 | * |
| 679282SNP | 7 | 43.89 |  |
| 550313SNP | 7 | 44.5 |  |
| 550281SNP | 7 | 44.78 |  |
| 318400SNP | 7 | 47.51 |  |
| 311850SNP | 7 | 47.8 |  |
| 379434SNP | 7 | 48.38 |  |
| 239841SNP | 7 | 48.66 |  |
| 191138SNP | 7 | 49.58 |  |
| 5760SNP | 7 | 52.77 |  |
| 9919222SNPCa7 | 7 | 53.34 |  |
| 9919209SNPCa7 | 7 | 53.63 |  |
| 10091989SNPCa7 | 7 | 54.2 |  |
| 10299185SNPCa7 | 7 | 55.08 |  |
| 10295151SNPCa7 | 7 | 55.37 |  |
| 10619273SNPCa7 | 7 | 56.54 |  |
| 10900908SNPCa7 | 7 | 57.11 |  |
| 11114257SNPCa7 | 7 | 57.4 |  |
| 11115193SNPCa7 | 7 | 57.69 |  |
| 11478260SNPCa7 | 7 | 58.66 | * |
| 11478235SNPCa7 | 7 | 58.99 | * |
| 11588670SNPCa7 | 7 | 59.65 |  |
| 11650065SNPCa7 | 7 | 60.24 |  |
| 11729772SNPCa7 | 7 | 60.52 |  |
| 12066122SNPCa7 | 7 | 60.81 |  |
| 12254261SNPCa7 | 7 | 61.68 |  |
| 12245815SNPCa7 | 7 | 61.96 |  |
| 12382714SNPCa7 | 7 | 62.54 |  |
| 12496588SNPCa7 | 7 | 62.82 |  |
| 12838539SNPCa7 | 7 | 64.29 |  |
| 13264019SNPCa7 | 7 | 64.58 |  |
| 13552548SNPCa7 | 7 | 65.16 |  |
| 13655331SNPCa7 | 7 | 65.45 |  |
| 13796332SNPCa7 | 7 | 66.41 | * |
| 13826671SNPCa7 | 7 | 66.75 | * |
| 13921996SNPCa7 | 7 | 67.39 |  |
| 14237195SNPCa7 | 7 | 67.69 | * |
| 14354183SNPCa7 | 7 | 67.99 |  |
| 14453020SNPCa7 | 7 | 68.28 |  |
| 14868089SNPCa7 | 7 | 69.14 |  |
| 15130009SNPCa7 | 7 | 69.43 |  |
| 15207222SNPCa7 | 7 | 70.01 |  |
| 15871224SNPCa7 | 7 | 70.9 | * |
| 16331943SNPCa7 | 7 | 71.78 | * |
| 16278384SNPCa7 | 7 | 72.07 | * |
| 16624369SNPCa7 | 7 | 73.54 |  |
| 16683872SNPCa7 | 7 | 74.42 |  |
| 16692287SNPCa7 | 7 | 74.73 | * |
| 17777009SNPCa7 | 7 | 75.35 |  |
| 21510487SNPCa7 | 7 | 77.13 |  |
| 20971505SNPCa7 | 7 | 77.41 |  |
| 47612773SNPCa7 | 7 | 77.7 |  |
| 24505616SNPCa7 | 7 | 78.27 | * |
| 39349254SNPCa7 | 7 | 79.43 | * |
| 35808365SNPCa7 | 7 | 80.59 | * |
| 35343268SNPCa7 | 7 | 81.16 |  |
| 35123198SNPCa7 | 7 | 81.44 |  |
| 34907066SNPCa7 | 7 | 82.61 |  |
| 34778980SNPCa7 | 7 | 83.36 |  |
| 34516785SNPCa7 | 7 | 83.73 |  |
| 316497SNP | 7 | 84.6 |  |
| 226279SNP | 7 | 84.88 |  |
| 121344SNP | 7 | 85.45 |  |
| 34347486SNPCa7 | 7 | 85.76 |  |
| 6038SNP | 7 | 86.37 |  |
| 34329154SNPCa7 | 7 | 86.94 | * |
| 33821320SNPCa7 | 7 | 87.54 | * |
| 33579255SNPCa7 | 7 | 88.15 | * |
| 33335353SNPCa7 | 7 | 88.72 |  |
| 33238492SNPCa7 | 7 | 89.67 |  |
| 32724178SNPCa7 | 7 | 90.3 |  |
| 32401257SNPCa7 | 7 | 91.16 |  |
| 32053990SNPCa7 | 7 | 91.73 |  |
| 31908269SNPCa7 | 7 | 92.02 |  |
| 31823923SNPCa7 | 7 | 93.24 |  |
| 31432061SNPCa7 | 7 | 95.41 |  |
| 30748150SNPCa7 | 7 | 97.82 |  |
| 30291103SNPCa7 | 7 | 98.41 |  |
| 30219717SNPCa7 | 7 | 99.16 |  |
| 30125610SNPCa7 | 7 | 99.53 |  |
| 30035475SNPCa7 | 7 | 99.81 |  |
| 29905308SNPCa7 | 7 | 100.57 | * |
| 29800599SNPCa7 | 7 | 100.94 |  |
| 29653982SNPCa7 | 7 | 101.56 |  |
| 29575582SNPCa7 | 7 | 101.87 |  |
| 29193078SNPCa7 | 7 | 102.16 |  |
| 29079072SNPCa7 | 7 | 103.03 |  |
| 79892SNP | 7 | 104.2 |  |
| 592237SNP | 7 | 104.79 |  |
| 28453350SNPCa7 | 7 | 105.66 |  |
| 28486280SNPCa7 | 7 | 106.56 |  |
| 21823738SNPCa7 | 7 | 164.62 | * |
| 16356599SNPCa8 | 8 | 0 | * |
| 16175458SNPCa8 | 8 | 0.31 | * |
| 15769714SNPCa8 | 8 | 0.93 | * |
| 15595102SNPCa8 | 8 | 2.72 | * |
| 15513692SNPCa8 | 8 | 3.29 | * |
| 15471398SNPCa8 | 8 | 3.86 | * |
| 14931313SNPCa8 | 8 | 4.43 | * |
| 14866458SNPCa8 | 8 | 5 | * |
| 14815387SNPCa8 | 8 | 6.46 | * |
| 14565208SNPCa8 | 8 | 7.03 | * |
| 332857SNP | 8 | 13.59 | * |
| 8268411SNPCa8 | 8 | 18.75 | * |
| 7997292SNPCa8 | 8 | 19.36 | * |
| 7756965SNPCa8 | 8 | 19.65 | * |
| 6951401SNPCa8 | 8 | 20.22 | * |
| 6263980SNPCa8 | 8 | 20.8 | * |
| 6030071SNPCa8 | 8 | 21.08 | * |
| 5994344SNPCa8 | 8 | 21.66 | * |
| 5965724SNPCa8 | 8 | 22.24 | * |
| 5883411SNPCa8 | 8 | 22.54 | * |
| 5483334SNPCa8 | 8 | 23.44 | * |
| 5031057SNPCa8 | 8 | 24.61 | * |
| 4955498SNPCa8 | 8 | 25.18 | * |
| 4810750SNPCa8 | 8 | 25.75 | * |
| 4725927SNPCa8 | 8 | 26.06 | * |
| 4640425SNPCa8 | 8 | 26.37 | * |
| 4303979SNPCa8 | 8 | 26.95 | * |
| 4177646SNPCa8 | 8 | 28.11 | * |
| 4097398SNPCa8 | 8 | 28.39 | * |
| 3765639SNPCa8 | 8 | 29.25 | * |
| 3666931SNPCa8 | 8 | 29.54 | * |
| 3602213SNPCa8 | 8 | 30.4 | * |
| 3531840SNPCa8 | 8 | 30.69 | * |
| 3402212SNPCa8 | 8 | 30.98 | * |
| 3247719SNPCa8 | 8 | 31.55 | * |
| 2961944SNPCa8 | 8 | 32.12 | * |
| 2742760SNPCa8 | 8 | 33.09 | * |
| 2527942SNPCa8 | 8 | 33.73 | * |
| 2392473SNPCa8 | 8 | 34.01 | * |
| 2309857SNPCa8 | 8 | 34.59 | * |
| 2271784SNPCa8 | 8 | 35.17 | * |
| 2171187SNPCa8 | 8 | 35.75 | * |
| 2088222SNPCa8 | 8 | 36.32 | * |
| 1845526SNPCa8 | 8 | 36.6 | * |
| 1615457SNPCa8 | 8 | 38.75 | * |
| 1481477SNPCa8 | 8 | 39.34 | * |
| 1379932SNPCa8 | 8 | 39.63 | * |
| 1176118SNPCa8 | 8 | 41.08 | * |
| 1063387SNPCa8 | 8 | 41.94 | * |
| 981649SNPCa8 | 8 | 42.23 | * |
| 969960SNPCa8 | 8 | 43.98 | * |
| 866999SNPCa8 | 8 | 44.56 | * |
| 802587SNPCa8 | 8 | 44.84 | * |
| 748147SNPCa8 | 8 | 45.13 | * |
| 737285SNPCa8 | 8 | 45.71 | * |
| 702632SNPCa8 | 8 | 46 | * |
| 724559SNPCa8 | 8 | 46.29 | * |
| 507908SNPCa8 | 8 | 46.86 | * |
| 635196SNPCa8 | 8 | 47.15 | * |
| 615632SNPCa8 | 8 | 47.43 | * |
| 441452SNPCa8 | 8 | 48.29 | * |
| 441551SNPCa8 | 8 | 48.58 | * |
| 331668SNPCa8 | 8 | 49.73 | * |
| 331624SNPCa8 | 8 | 50.02 | * |
| 215154SNPCa8 | 8 | 50.3 | * |
| 31311SNPCa8 | 8 | 50.59 | * |

^a^Markers with segregation distortion are denoted with the “*” sign.

# Supplementary Table 5. Test statistics for the six models with MSD values used for association analysis.

| Model | Kinship (K) | PC2 (25%)+K | PC3 +K | PC4+K | PC5 (50%)+K | Admixture(Q2) +K | Admixture(Q4)+K |
| --- | --- | --- | --- | --- | --- | --- | --- |
| MSD | 1.98E^-06^† | 6.71E^-05^ | 4.17E^-05^ | 1.22E^-04^ | 1.02E^-04^ | 6.71E^-05^ | 6.34E^-05^ |

† Least mean square deviation (MSD) values among all models selected for GWAS. K is Kinship and PC is Principal components (PC2 – PC5) explaining 25% to 50% variance.


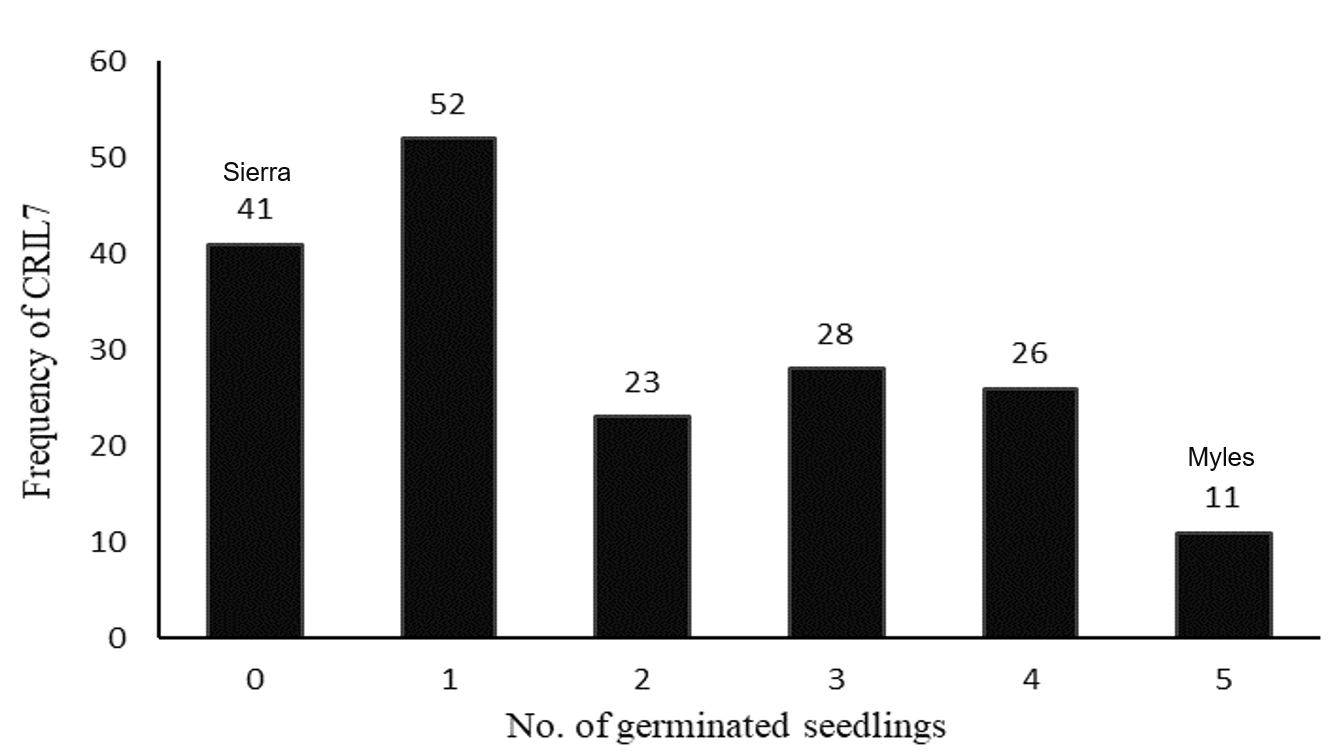


A


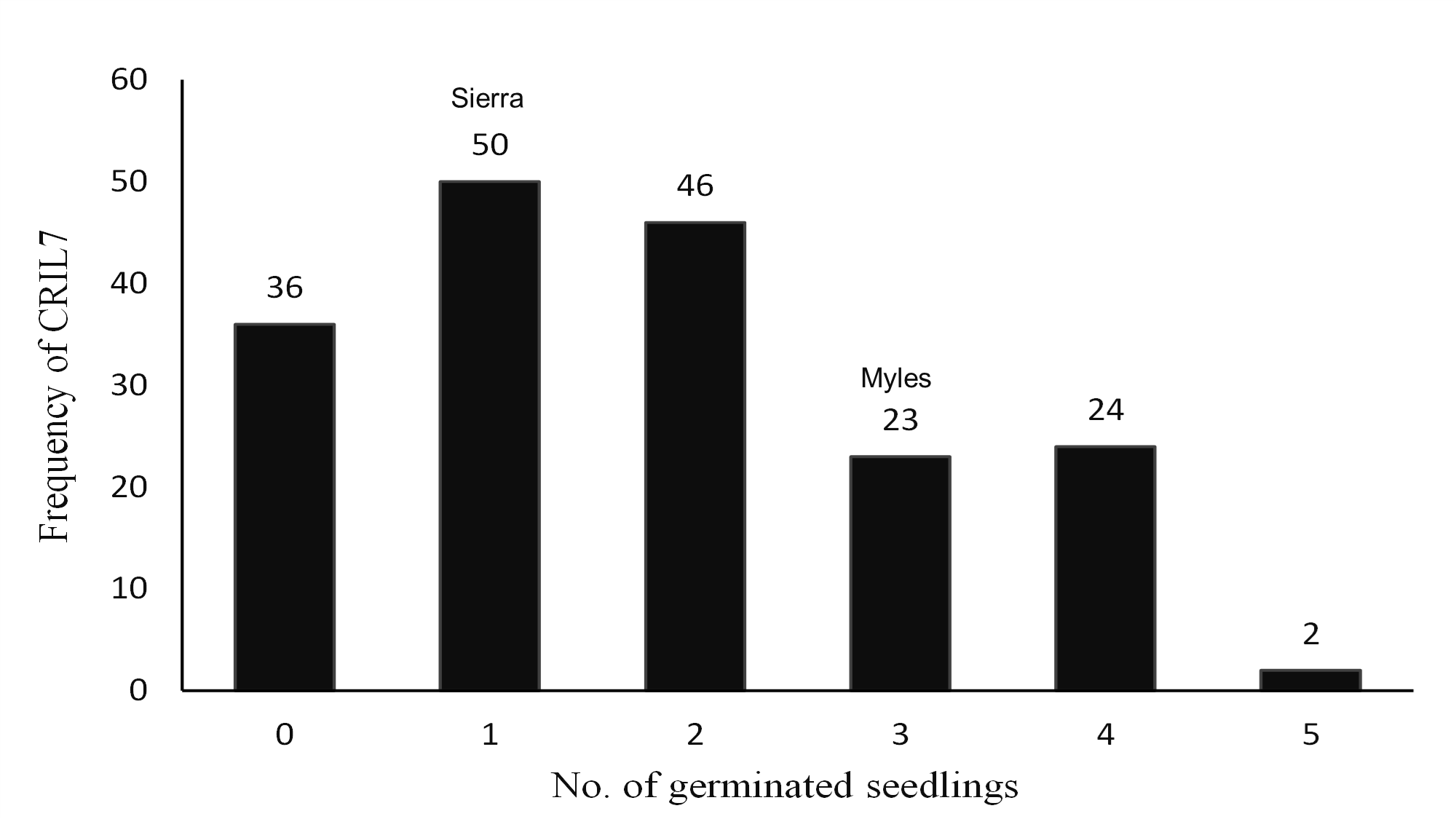


B

Supplementary Fig 1. Frequency distributions of germinated seedlings in the CRIL-7 population along with parents and controls. A is frequency distribution of CRIL7 in experiment 1. B is frequency distribution of CRIL7 in experiment 2. X-axis represents number of germinated seedlings from score 0 (no germination) to score 5 (germination of all seeds). Numbers on each bar represents total number of CRIL7 lines for the given score. Myles is the resistant and Sierra is the susceptible control.


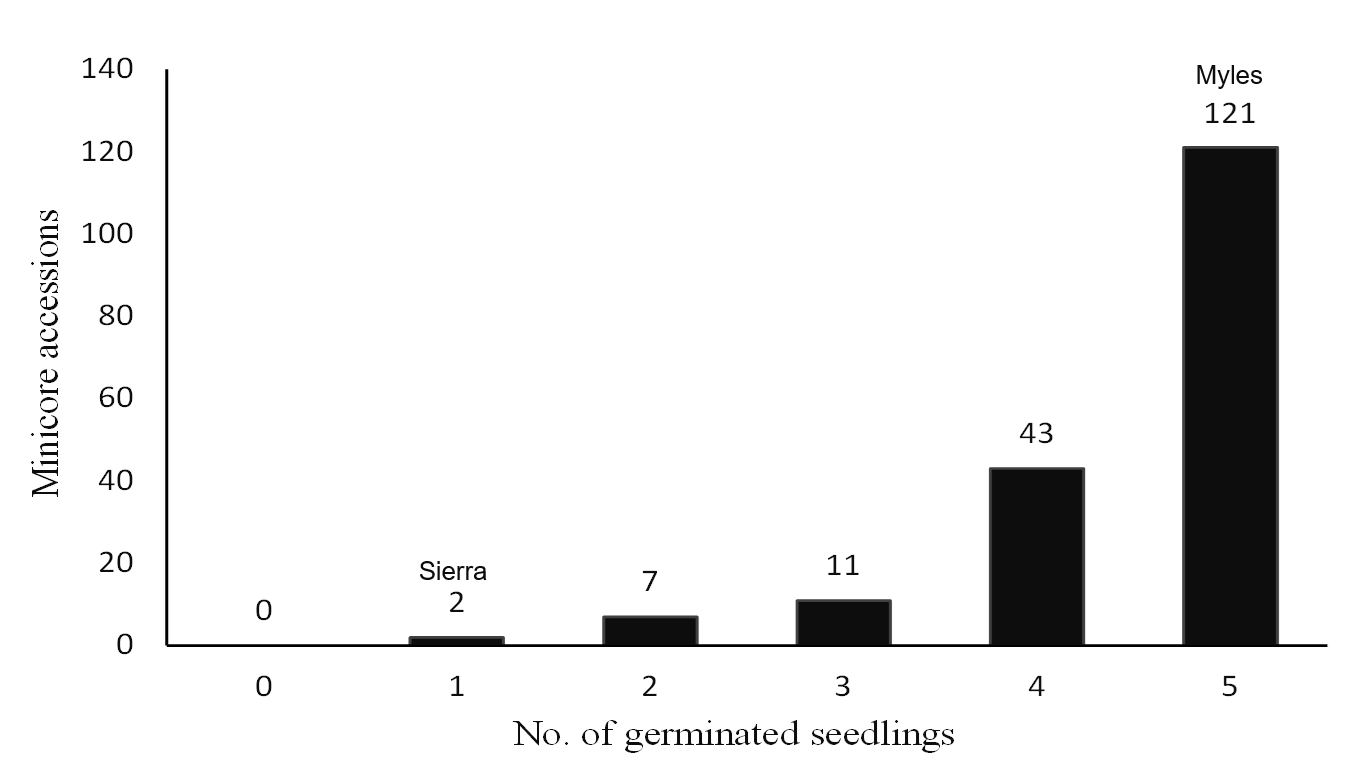


A


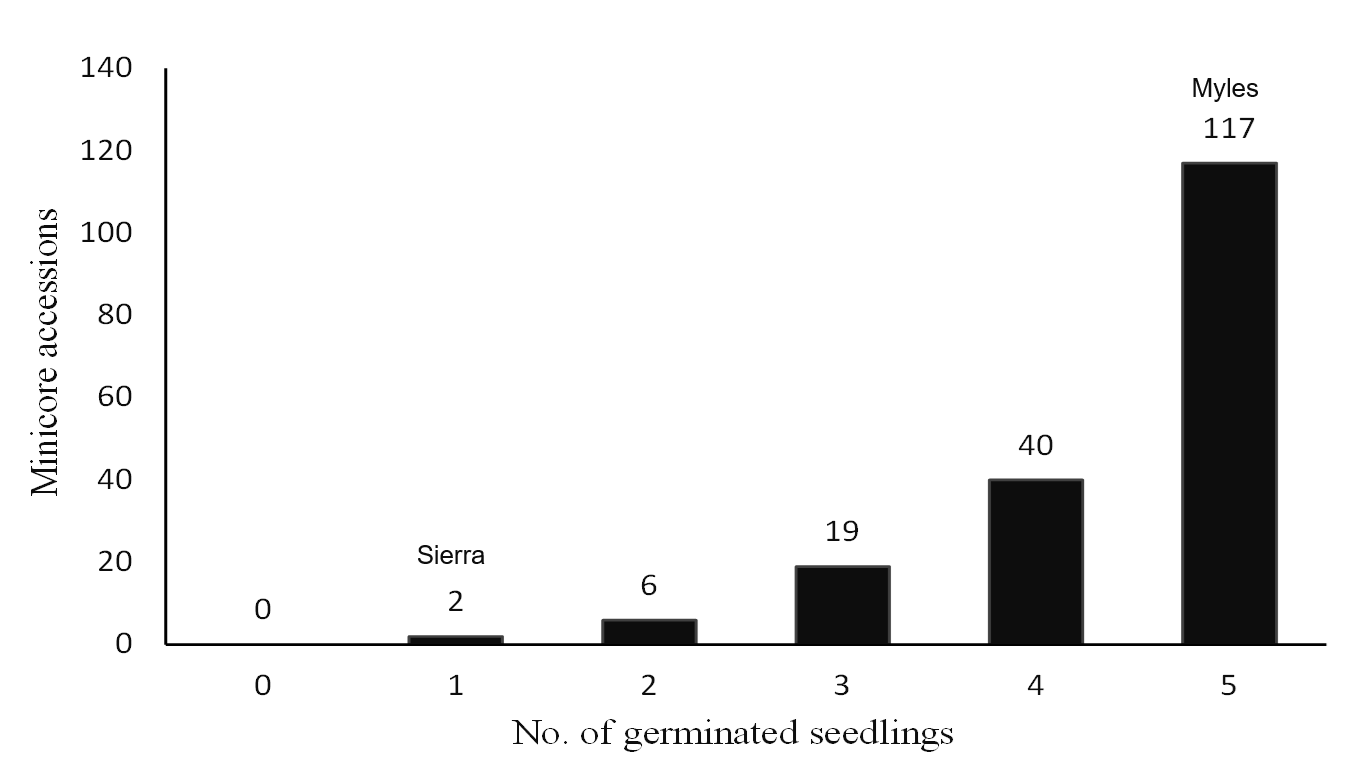


B

Supplementary Fig 2. Frequency distributions of germinated seedlings among the mini-core accessions along with controls. (A) is frequency distribution of mini-core accessions in experiment 1. (B) is frequency distribution of mini-core accessions in experiment 2. X-axis represents number of germinated seedlings from score 0 (no germination) to score 5 (germination of all seeds). Numbers on each bar represents total number of accessions for the given score. Myles is the resistant and Sierra is the susceptible control.

Supplementary Fig 3. Genetic map of chickpea CRIL7 [*C. reticulatum* (PI 599072) x *C. arietinum* (FLIP 84-92C)] population. The map includes 8 linkage groups ( LG1, LG2, LG3, LG4, LG5, LG6, LG7 and LG8). The black bars on the linkage groups represent 1029 SNP markers and red bars on LG4 and LG8 represent position of the QTLs. The crudeness black bar represents closely spaced markers.


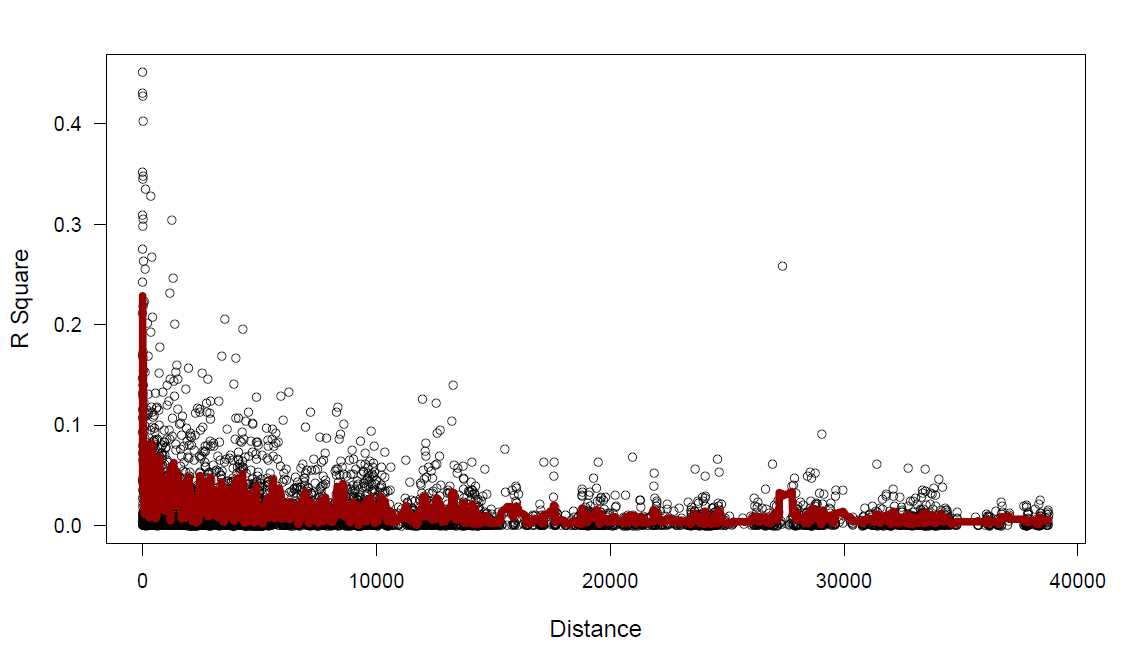


Supplementary Fig 4. LD extent measured in chickpea genotypes using 302,902 SNP markers. Horizontal axis corresponds to physical distance (bp), vertical axis corresponds to Linkage Disequilibrium (LD) between SNP markers measured as r^2^. The red curves are the trend of LD extent fitted using the mutation-drift-equilibrium model.
